# Predicting and explaining the impact of genetic disruptions and interactions on organismal viability

**DOI:** 10.1093/bioinformatics/btac519

**Published:** 2022-07-21

**Authors:** Bader F Al-Anzi, Mohammad Khajah, Saja A Fakhraldeen

**Affiliations:** Food and Nutrition Program, Kuwait Institute for Scientific Research, Safat 13109, Kuwait; Systems and Software Development Department, Kuwait Institute for Scientific Research, Safat 13109, Kuwait; Ecosystem-based Management of Marine Resources Program, Kuwait Institute for Scientific Research, Safat, 13109, Kuwait

## Abstract

**Motivation:**

Existing computational models can predict single- and double-mutant fitness but they do have limitations. First, they are often tested via evaluation metrics that are inappropriate for imbalanced datasets. Second, all of them only predict a binary outcome (viable or not, and negatively interacting or not). Third, most are uninterpretable black box machine learning models.

**Results:**

Budding yeast datasets were used to develop high-performance Multinomial Regression (MN) models capable of predicting the impact of single, double and triple genetic disruptions on viability. These models are interpretable and give realistic non-binary predictions and can predict negative genetic interactions (GIs) in triple-gene knockouts. They are based on a limited set of gene features and their predictions are influenced by the probability of target gene participating in molecular complexes or pathways. Furthermore, the MN models have utility in other organisms such as fission yeast, fruit flies and humans, with the single gene fitness MN model being able to distinguish essential genes necessary for cell-autonomous viability from those required for multicellular survival. Finally, our models exceed the performance of previous models, without sacrificing interpretability.

**Availability and implementation:**

All code and processed datasets used to generate results and figures in this manuscript are available at our Github repository at https://github.com/KISRDevelopment/cell_viability_paper. The repository also contains a link to the GI prediction website that lets users search for GIs using the MN models.

**Supplementary information:**

Supplementary data are available at *Bioinformatics* online.

## 1 Introduction

Advancements in sequencing technology have allowed the sequencing of entire genomes in a very short time (Hu *et al.*, 2021). But it is still challenging to experimentally determine the contribution of each individual gene within these genomes to organismal viability. This problem is compounded by the fact that the impact of many of these genes often depends on the genetic background of the target individual due to a phenomenon known as Genetic Interaction (GI) (Collins *et al.*, 2010; Costanzo *et al.*, 2010; Costanzo *et al.*, 2016; Costanzo *et al.*, 2019) (Summary of the different types of GIs is provided in Supplementary Fig. S1).

Computational and mathematical models are becoming an essential component for analyzing large biological data sets, such as the one generated by genomics, since they enable the simulation of thousands of manipulations, thereby reducing the number of required laboratory experiments to a more manageable set of key validations. Indeed, significant effort has been invested in developing models that can either predict essential genes, characterized by a lethal phenotype upon deletion (Campos *et al.*, 2019; Campos *et al.*, 2020; Cheng *et al.*, 2014; Gabriel del Rio, 2009; Li *et al.*, 2012; Luo and Wu, 2015; Zhang *et al.*, 2016), or identify novel negative GIs (Al-Aamri *et al.*, 2019; Benstead-Hume *et al.*, 2017; Benstead-Hume *et al.*, 2019; Chipman and Singh, 2009; Paladugu *et al.*, 2008; Srivas *et al.*, 2016; Wong *et al.*, 2004; Young and Marcotte, 2017; Yu *et al.*, 2016). However, analysis of the outputs of many existing models reveals some limitations (for review, see Madhukar *et al.*, 2015). First, in the majority of cases, the main evaluation metric is the Area Under the Receiver Operating Characteristic Curve (AUC-ROC) (Al-Aamri *et al.*, 2019; Benstead-Hume *et al.*, 2019; Gabriel del Rio, 2009; Alanis-Lobato, 2013; Li *et al.*, 2012; Mistry *et al.*, 2017; Wong *et al.*, 2004; Wu *et al.*, 2014), which measures the ability of a binary classifier to distinguish one class from another (e.g. lethal versus viable) at different false positive rates (Bradley, 1997). Unfortunately, this method has been shown to overestimate model performance when the classes being evaluated are imbalanced in size (Davis and Goadrich, 2006; Saito and Rehmsmeier, 2015). Indeed, a considerable portion of biological datasets exhibits such class imbalance. For example, in budding yeast, negative GIs comprise only about 0.8% of all double mutant combinations. Some researchers compensate for this issue by explicitly balancing conditions, either by over-sampling (e.g. generating a higher count of exceptional conditions) or under-sampling (e.g. eliminating frequent conditions) (Benstead-Hume *et al.*, 2019; Campos *et al.*, 2019; Li *et al.*, 2012; Wu *et al.*, 2014). However, applying such artificial balancing schemes changes the target statistical distribution, resulting in an overestimation of model performance. Second, most published models are limited to only binary predictions *(*e.g. essential versus non-essential (Campos *et al.*, 2019; Campos *et al.*, 2020; Li *et al.*, 2012; Luo and Qi, 2015; Luo and Wu, 2015; Mistry *et al.*, 2017; Zhang *et al.*, 2016), or negative GIs versus all other interactions (Benstead-Hume *et al.*, 2019; Li *et al.*, 2012; Wang *et al.*, 2015; Wu *et al.*, 2014). Since most diseased states are not binary, but rather exhibit variations in their outcome, a model that can predict more than two states would be more realistic and biologically relevant. Nevertheless, binary classifications are still helpful in understanding which features lead to lethality and which do not. Third, a considerable portion of proposed models is of the black box type, in which the internal workings of the model are not made explicit. Indeed, even in cases where the authors assess the effect of various input features on model performance, such attempts still do not explain how the model uses the input features to produce its predictions, i.e. they are still black box models (Benstead-Hume *et al.*, 2019; Li *et al.*, 2012; Wu *et al.*, 2014). Finally, most of these studies do not provide comparisons between the proposed, and often elaborate, computational model to simpler baseline models.

In this article, we used custom feed-forward Neural Networks (NNs) (G**é**ron, 2019; Goodfellow *et al.*, 2016) to predict the impact of single, double and triple gene knockout on growth of the budding yeast *Saccharomyces cerevisiae* using multiple molecular characteristics of proteins encoded by the genome. Feature selection was then performed on these models to create refined models composed of the smallest set of input features capable of achieving comparable performance to the full NN models. Additional constraints that allowed for direct interpretation of these models’ internal structures resulted in simpler open-box multinomial regression (MN) models (for model development see Supplementary Fig. S2). All of these models achieved relatively high performance when evaluated by several metrics that are appropriate for imbalanced datasets, such as Balanced Accuracy (BA), which calculates the average of the per-class accuracies (Brodersen, 2010; Velez, 2007) and confusion matrix, which shows a complete picture of classifier performance at a given decision threshold by counting the frequency of all predicted versus observed class combinations (Ting, 2017). Furthermore, even though the frequently used AUC-ROC metric is not appropriate for unbalanced datasets, the performance of these MN models in per-class classification (lethal versus everything else or interacting versus neutral) was superior to the null model and was also better than the reported performance of previously published models (Brodersen, 2010; Ting, 2017; Velez, 2007). Finally, we examined the utility of these models in other organisms such as the fission yeast *Schizosaccharomyces pombe*, the fruit fly *Drosophila melanogaster* and humans *Homo sapiens*. In these organisms, all MN models showed considerable utility in predicting the impact of single and double genetic disruptions on viability.

To summarize, we’ve shown that simple MN models can easily handle tasks that were tackled previously with ML approaches. This finding is analogous to the well-known work of Ba and Caruana (Lei Jimmy Ba, 2014) where the authors found that simple shallow NNs can achieve comparable accuracy to multi-layered convolutional NNs, with the appropriate training algorithm.

## 2 Results

### 2.1 A mathematical model predicting the impact of single gene removal in budding yeast

Genome-wide bioinformatic datasets from budding yeast were used to generate target gene input features (Breitkreutz *et al.*, 2010; Gavin *et al.*, 2002; Ho *et al.*, 2002; Oughtred *et al.*, 2019; The Gene Ontology, 2019; Venters *et al.*, 2011). Overall, close to 300 input features that are classified into seven broad categories were used. The yeast single mutant knockout dataset was split into development and testing sets. All model development described below was performed on the development set via repeated cross-validation, and the final evaluation was performed on the test set. In all cases, models are trained with weighted categorical cross-entropy, which penalizes the models if they fail to assign a high probability to the true class. For details regarding computational models, input and output datasets, training and testing dataset splits, cross-validation and hyperparameter optimization (Supplementary Table S1, sheet 1), please see Supplementary Materials and Methods.

As stated earlier, one of our main arguments in this article is that an appropriate metric for evaluating model performance under class imbalance should be used. We primarily rely on BA, which, for binary classification, is defined mathematically as follows:

(1) BA=TPR+TNR2 

where TPR and TNR are the true positive and negative rates of the model, respectively. The TPR is the proportion of positively labeled examples that the model predicted, and the TNR is the proportion of negatively labeled examples that the model predicted. BA ensures that a model can’t simply do well by always predicting the majority class (e.g. saying no all the time) and that a model with no predictive power would have a BA of 0.5. The BA is extended to handle multi-class classification as follows:

(2) BA=1K∑c=1KfccNc  

where K is the number of classes, $N_{c}$ is the number of examples in class c and $f_{cc}$ is the number of correctly identified examples of class c. Again, this formulation penalizes models that perform poorly on the minority classes and assigns a BA of $\frac{1}{K}$ for models with no predictive power (so for three-way classification, a null model would achieve BA=0.333).

**Fig. 1.**
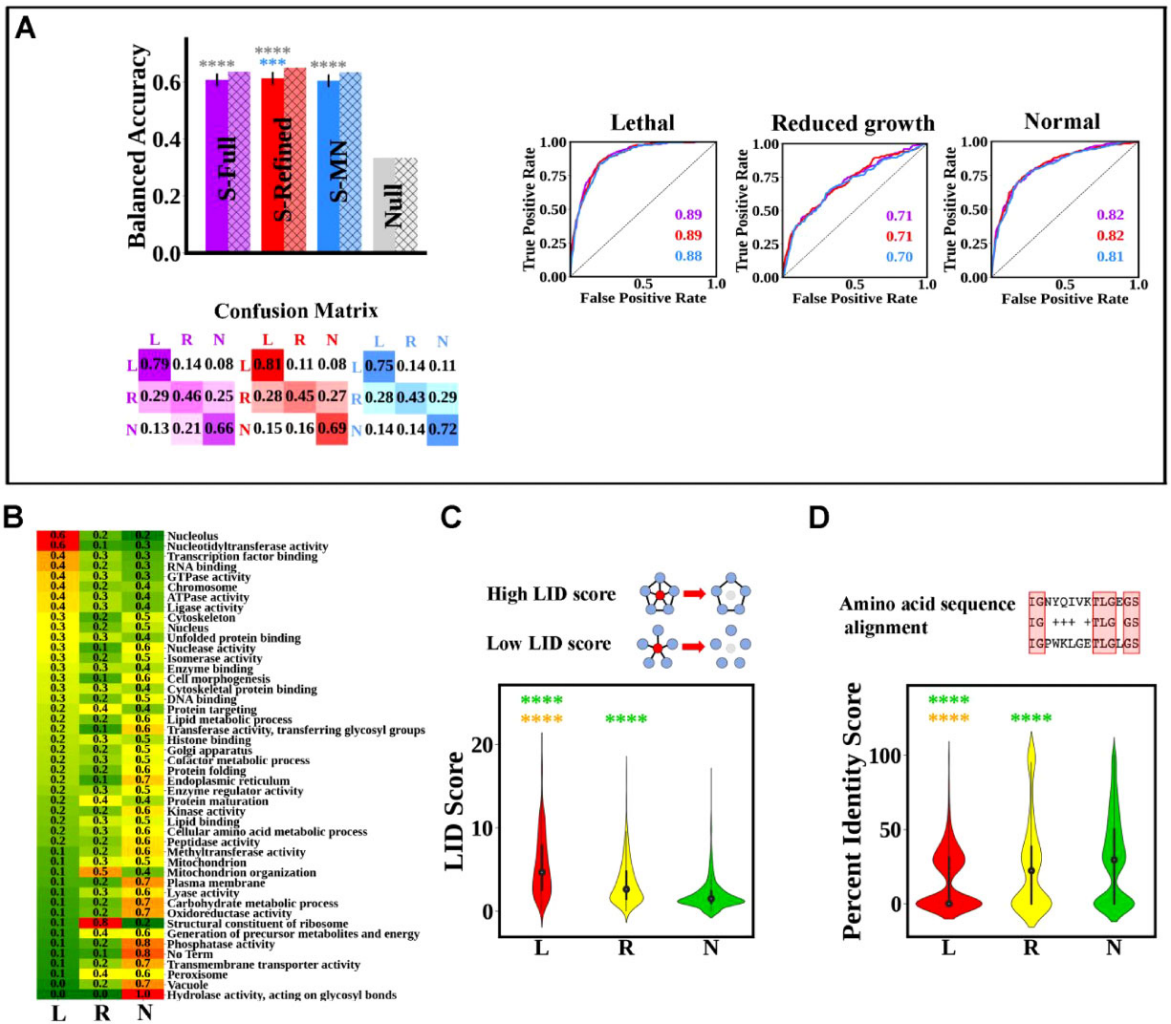
Performance of computational models when predicting the impact of single gene disruption on budding yeast viability. (**A**) Models’ performance as measured by overall BA on both the development dataset (solid color) and test dataset (hatched color), confusion matrices on the test dataset and per-class ROC on the test dataset (with the corresponding AUC-ROC values). The purple, red, blue and gray colors correspond to the S-Full, S-Refined, S-MN and null models, respectively. Error bars in the development set BA plots (solid color) correspond to SDs. The lack of error bars on test dataset BA results (hatched color) is due to the models being tested on a single withheld test dataset. Asterisks represent Bonferroni-corrected *P*-values, *P* < $\frac{0.05}{C}$ (*), *P* < $\frac{0.01}{C}$ (**), *P* <$\;\frac{0.001}{C}$ (***), and *P* <$\frac{0.0001}{C}$ (****), where *C* = 6, and asterisk colors correspond to the models being compared. (**B** and **C**) Differences in the distributions of input features used in the S-Refined and S-MN models across the three single mutant fitness classes. (B) A heatmap representing the prevalence of a given sGO term in the lethal (L), reduced growth (R) and normal growth (N) output classes. The heatmap is sorted along the prevalence in the L class. The values inside the cells correspond to the class distribution for each term. (C and **D**) Violin plots showing the distribution in each output class of the LID and percent amino acid identity, with an illustration of these input features in the upper portion of each panel. The circle in each violin corresponds to the median value and the thick black line corresponds to the interquartile range (middle 50% of observations). Asterisks in (B) and (C) represent the Bonferroni-corrected reliability of the Kruskal–Wallis test with *C* = 3 (A color version of this figure appears in the online version of this article.)

A feed-forward NN was used to construct the initial black-box model (G**é**ron, 2019; Goodfellow *et al.*, 2016) (Supplementary Fig. S3A). This single gene fitness full model (S-Full), which uses all input feature sets, performs well when identifying lethal and normal class single mutants and performs moderately when predicting the reduced growth class on the development dataset (Fig. 1A, solid purple).

Feature selection was performed on the development dataset by enumerating all possible combinations of input feature sets generating 127 models, followed by selecting the model with the smallest set of input features that achieves the highest prediction performance (Supplementary Table S2). This produces a single gene fitness refined model (S-Refined) that uses three types of input features: First, the basic 45 slim Gene Ontology (sGO) terms shared with other organisms that broadly describe the biological processes of the target protein, its localization in cellular compartments and its molecular activity (The Gene Ontology, 2019). The second feature is the LID centrality, which is the density of connections remaining between the direct neighbors of the protein encoded by the target gene after its removal in the protein-protein interaction network (PPI) (Luo and Qi, 2015) and is considered to be a proxy for the level of internal connectedness of the target protein complex. The third feature is the sequence percent identity, which is the level of sequence homology between the target protein and its closest BlastP match in the yeast genome. The relevance of these selected input features was confirmed by assessing their statistical differences in the different output classes (Fig. 1B–D).

It is worth noting that the most prevalent sGO terms in the lethal class are terms for processes that are essential for survival, meaning they are required under optimal growth conditions (Zhang and Ren, 2015), such as nucleolus (essential for rRNA production and processing; Alberts B, 2002), nucleotidyltransferase activity (necessary for DNA repair; Yuan Liu, 2007) and transcription factor binding (necessary for RNA synthesis; Babu, 2004). For genes whose mutation was predicted to result in normal growth, however, the opposite distribution pattern was observed, with the normal class being enriched with sGO terms such as hydrolase activity, which acts on glycosyl bonds and is required to break complex carbohydrates and amino acid transport. Both of these are less essential as budding yeast, which is capable of synthesizing most amino acids *de novo*, and can grow in media supplemented with simple monosaccharides (i.e. dextrose) as an energy source (Dever and Hinnebusch, 2005).

Using this minimum set of input features, we generated a simpler interpretable multinomial regression model (S-MN). This model has one equation per output class (i.e. lethal and reduced growth), each specifying the log odds of the probability of the corresponding class relative to a reference normal growth class. All equations follow the same structure:

(3) LOG⁡p xp n=βox+β1xLID+ β2xPident+ ∑iβi+2xsGOi 

The S-MN model equations are defined in terms of log odds with respect to the neutral class for the purpose of interpreting the model coefficients only. Thus, similar to the NN models, it still must distinguish between all classes in order to achieve high performance.

In Equation (3), *x* denotes the lethal and reduced growth classes, $p_{x}$ represents the probability of the corresponding class and $p_{n}$ represents the probability of the normal growth class. The input features are the same as the refined model: The LID, the percent identity to the closest BlastP match and the sGO terms. Each feature has a coefficient $\beta_{ix}$ which quantifies its contribution to the prediction. Those coefficients are free parameters and are learned from the training set via stochastic gradient descent (see Supplementary Materials and Methods). On the development dataset, the S-MN model achieved similar performance in terms of overall BA to the S-Refined and S-Full models (Fig. 1A, solid blue). The coefficient values can be easily interpreted, for example, the removal of genes with sGO term Ligase activity increases the odds of a lethal phenotype by a factor of 2.31. For a full list of the three-way S-MN classifier coefficient values, please see Supplementary Table S3, sheet 1.

Finally, on the withheld test dataset, the S-Full, S-Refined and S-MN models achieved similar prediction performance, indicating that our methodology did not overfit (Chicco, 2017) (Fig. 1A, hatched purple, red and blue, respectively). We note that the performance on the withheld test set is higher than on the development set which might be counter-intuitive. However, since the withheld test set was randomly sampled from the original dataset, it could be the case that it just happened to be slightly easier than the development set by chance (recall that the results on the development set are obtained by averaging over 50 replications). All in all, all models achieve BA > 0.6, which is double the chance rate of 0.33, and shows that they are not merely predicting the majority normal class all the time.

### 2.2 The D-MN model can predict double-gene GIs in budding yeast

An approach similar to the one employed to generate the single mutant fitness models was used to construct a model capable of predicting GIs in budding yeast. However, additional input features were considered, such as impact of removing each gene in the target gene pair on cell viability, and pairwise gene features, such as the shortest path length between the proteins encoded by the target gene pair. The NN architecture was also modified to account for the symmetry between genes in a pair (i.e. pair A-B is the same as B-A). Specifically, a Siamese NN architecture (Jane Bromley, 1993) containing three sub-NNs modules was used: The first two modules were identical and contained sub-NNs that process input feature sets of either gene in a given gene pair while the third module contained a sub-NN that processes pairwise feature sets that jointly characterize both genes in the pair (Supplementary Fig. S3B). All model development was performed similarly to the single mutant fitness models stated above (please see Supplementary Materials and Methods and Supplementary Table S1, sheet 2).

The double gene GI full model (D-Full) reliably, but modestly, outperformed the control null baseline model on the test dataset (Supplementary Fig. S5A, purple). However, it is known that budding yeast genes can remain functionally inactive under normal growth conditions and only become activated under specific sets of culture conditions (e.g. high salt, high temperature, growth on non-fermentable sugars, etc.) (Imbeault *et al.*, 2008; Raja *et al.*, 2017; Waples *et al.*, 2009). GIs between such genes will be undetectable under normal growth conditions, given that the relevant signaling pathways are not active to begin with and thus cannot be suppressed or enhanced. To overcome this issue, a hybrid dataset was created by combining the neutral interactions in the Costanzo *et al.* (2016) dataset with GI datasets from BioGRID (Imbeault *et al.*, 2008; Raja *et al.*, 2017; Waples *et al.*, 2009; See Supplementary Materials and Methods).

**Fig. 2.**
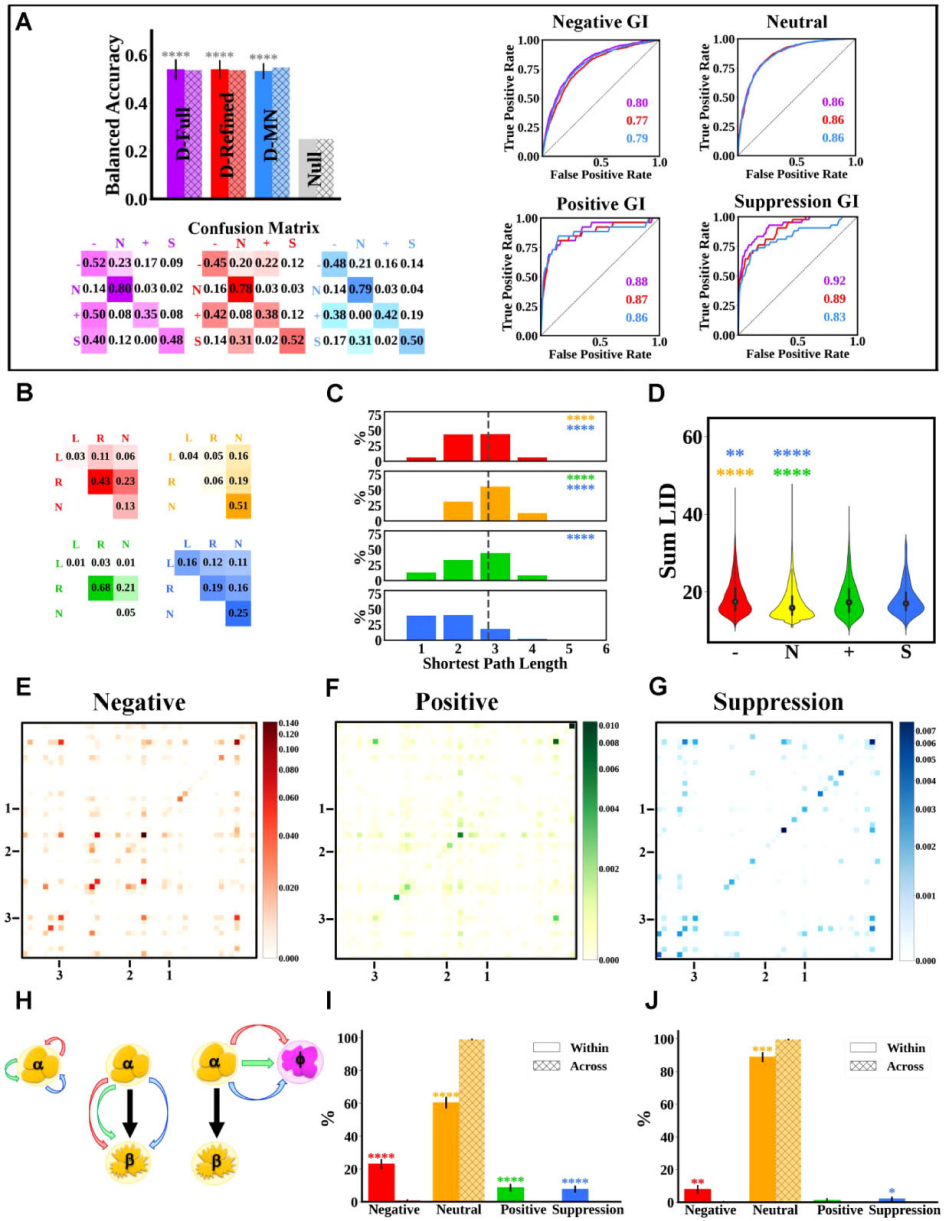
Performance of computational models when predicting double gene knockout GIs in the budding yeast. (**A**) Models’ performance on the development and test sets as measured by overall BA, confusion matrices and per-class AUC-ROC. The purple, red, blue and gray colors correspond to the D-Full, D-Refined, D-MN and null models, respectively. In all panels, the output classes are negative (−), neutral (N), positive (+) and suppression (S). Statistical analysis (error bars and asterisks) is similar to Figure 1 with the asterisk color reflecting the model being compared. (**B**–**G**) Differences in the distributions of input features used in the D-Refined and D-MN models across the four double gene knockout GI classes. (B) Matrices showing rows and columns which correspond to the single mutant fitness of the first and second gene in a pair: lethal (L), reduced growth (R) and normal (N). Each cell in a table shows the relative frequency of a particular pair of single mutant fitness classes in a GI class. (C) The distributions of the shortest path lengths separating proteins encoded by the target gene pairs in each GI class as compared to the average shortest path length between any protein pair in the PPI network (gray dashed line). (D) Violin plot showing the distribution of sum LID of proteins encoded by the target gene pairs in each of the four output classes. Statistical tests and symbols in the violin plot are similar to Figure 1. (E–G). The prevalence of sGO term pairs in a given GI class in matrix format. In each matrix, the cell color intensity indicates how often the sGO term of gene A (rows) appears with the sGO term of gene B (columns). The tick marks correspond to specific sGO terms: kinase activity (1), protein targeting (2) and chromosome (3). For a magnified version which includes the full labels of the sGO terms, see Supplementary Figure S6. (**H**) Cartoon of possible GIs combinations that can occur between genes in molecular complexes and pathways. The arrow represents negative (red), positive (green) and suppression (blue) GIs between genes that belong to the same molecular complex (left panel), between genes that belong to different molecular complexes (α and β) but are on the same pathway as indicated by the black arrow, and between genes that belong to different molecular complexes and pathways (right panel). For each complex (**I**) and pathway (**J**), we compute the distributions of within-complex (solid bars) and across-complex (textured bars) interactions over the four output classes. Asterisks correspond to *P*-value levels of two-sided *t*-test (A color version of this figure appears in the online version of this article.)

On the development portion of this hybrid dataset, the D-Full model performed well with the only exception being the positive GI class (Fig. 2A, purple). This difference in performance between the two datasets can be explained by the stricter criteria of inclusion in the hybrid datasets (at least three reported publications).

The same feature selection procedure used on the single mutant fitness model was applied to determine the smallest number of input feature sets that can be used without loss of predictive performance on the development portion of the dataset (see Supplementary Table S4). This produced a double gene GI refined model (D-Refined) with comparable performance to the D-Full model (Fig. 2A, solid red). The D-Refined model takes only four input feature sets: the LID centrality score of the proteins encoded by the target genes, the experimentally derived impact of removing the target genes on cell viability, the shortest path length between the proteins encoded by the target gene pairs, and the sGO terms associated with the genes in the gene pair. Analysis of selected input features indicates that the frequent mislabeling of positive GIs as negative GIs is most likely due to both GI classes being enriched in gene pairs with reduced growth phenotypes in single mutants (Fig. 2B) and have statistically insignificant differences in the distributions of shortest path length and LID scores (Fig. 2C and D, respectively). This is unlike the suppression and neutral GI classes, in which a clear and statistically difference between the negative and positive GI classes with respect to all three input features is observed (Fig. 2B–D, blue).

The above analysis indicates that an enrichment in interacting gene pairs occurs if they encode proteins that are part of the same molecular complex, since proteins within such context are expected to share a significant number of sGO terms, to be in close contact with one another and have a high LID score. Such a possibility is supported by previously published literature (Bandyopadhyay *et al.*, 2008; Bellay *et al.*, 2011; Costanzo *et al.*, 2016). The analyses from these published works were further expanded in order to distinguish between interactions within complexes from those within pathways (for data processing and list of selected molecular complexes and pathways please see Supplementary Material and Methods and Supplementary Table S5). Our analysis indicates that less than 5% of gene pairs that encode proteins that are not part of the same molecular complex or pathway show GIs with each other. This proportion significantly increases if the gene pair encodes proteins that are part of the same complex or pathway (40% and 20%, respectively; Fig. 2H–J).

Using the minimum set of input features required for making good predictions, we created a simpler open box double gene GI predicting MN model (D-MN) which has three equations, each specifying the log odds of the probability of the corresponding class (negative, positive and suppression) relative to a ‘reference’ neutral class. All equations followed the same structure:

(4) $$\mathrm{LOG}\left(\frac{p_{\;x}}{p_{\;n}}\right)=\phi_{0x}+\phi_{1x}\left(LID^{A}+LID^{B}\right)+\phi_{2x}LL+\phi_{3x}LR+\phi_{4x}LN+\phi_{5x}RR+\phi_{6x}RN+\phi_{7x}NN+\phi_{8x}SPL+\sum_{i}\phi_{\left(i+8\right)x}\left(sGO_{i}^{A}+sGO_{i}^{B}\right)$$

Similar to the S-MN model, the D-MN model equations are defined in terms of log odds with respect to the neutral class for the purpose of interpreting the model coefficients only.

In the equation, *x* denotes the negative, positive and suppression classes and $p_{x}$ represents the probability of the corresponding class (with $p_{n}$ representing the probability of the neutral class). The input features are as follows: the sum of LID scores of the two genes $\left(LID^{A}+LID^{B}\right)$, all possible combinations of single mutant fitness readings of the two genes (both lethal LL, one lethal and one reduced LR, one lethal and one normal LN, both reduced RR, one reduced and one normal RN, and both normal NN), the shortest path length between the genes (SPL), and finally whether both genes share the same sGO terms or if either of them has a given sGO term ($sGO_{i}^{A}+sGO_{i}^{B}$). The coefficients $\phi_{ix}$ correspond to weights imposed on the input features, which, as with the S-MN model, are free parameters and are learned via stochastic gradient descent. Since these coefficients are indexed by *x*, there is a separate set of coefficients for each of the GI classes. The D-MN performs similarly to the D-Refined model on the development dataset (Fig. 2, solid blue). For a full list of the four-way D-MN model coefficient values, see Supplementary Table S6, sheet 1 which can be easily interpreted as with the S-MN model.

Finally, on the withheld test dataset, the D-Full, D-Refined and D-MN models achieved very similar prediction performance (Fig. 2, hatched purple, red and blue, respectively) with a BA of about 0.55, which is more than double the chance rate of 0.25, indicating that despite the significant imbalance in the budding yeast GI dataset (almost 7 000 000 neutral versus about 23 000 GIs), the models are able to distinguish the classes from each other (except for the positive class, which is often confounded with the negative class).

### 2.3 The T-MN model can predict negative GI of a triple gene knockout in budding yeast

An extensive dataset reporting triple gene knockout manipulation covering 310 genes in budding yeast was published (Kuzmin *et al.*, 2018). However, this dataset only reports the negative and neutral phenotypes with the positive and suppression phenotypes being excluded by the authors due to low signal-to-noise ratio. Despite this caveat, the dataset remains worthy of examination.

Models predicting the impact of triple gene knockout on budding yeast viability were constructed using a similar approach to the one used to generate the D-MN model (Supplementary Fig. S3C). For details regarding computational models, input and output datasets, training and testing dataset splits and cross-validation, please see Supplementary Materials and Methods.

**Fig. 3.**
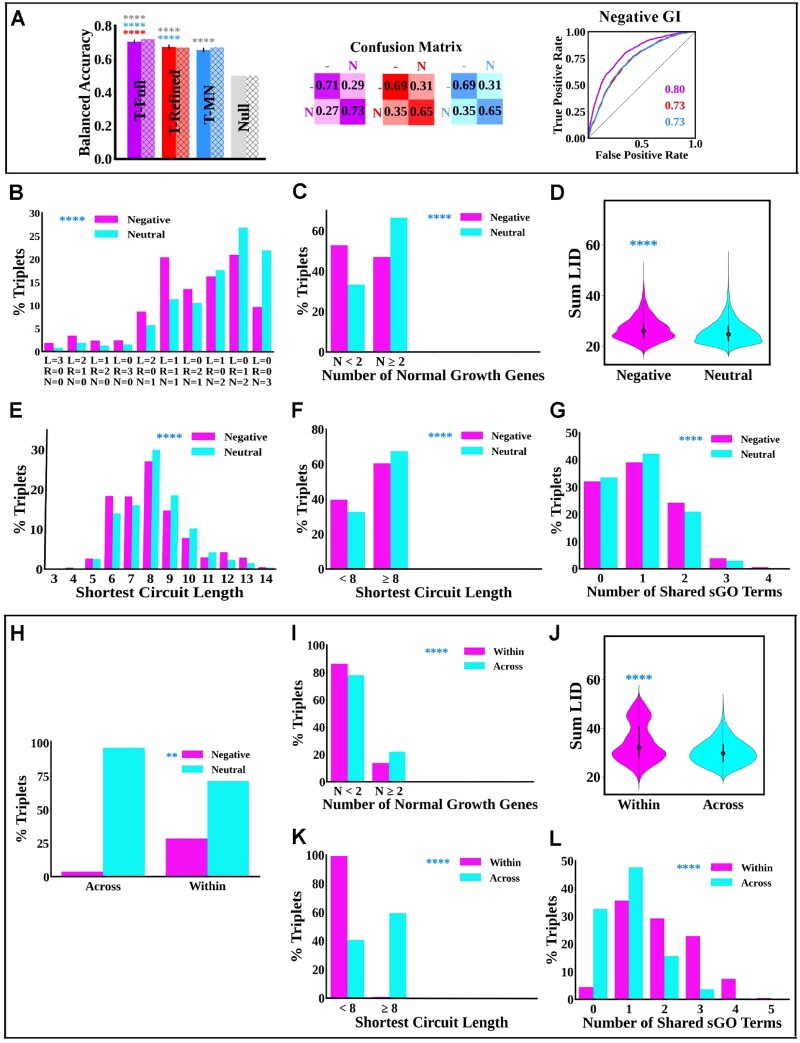
Performance of computational models predicting triple gene knockout negative GIs in the budding yeast. (**A**) Models’ performance on the development and test sets as measured by the same metrics in Figures 1 and 2. The purple, red, blue and gray colors correspond to the T-Full, T-Refined, T-MN and null models, respectively. In all panels, the output classes are negative (−) and neutral (N). Statistical analysis (error bars and asterisks) is similar to Figure 1. (**B**–**G**) Differences in the distributions of the input features used in the T-Refined model in the negative (magenta) and neutral (cyan) triple gene knockout GI classes. (B) Shows the percentages for each possible combination of single mutant fitness readings. Each tick along the *x*-axis shows the number of lethal (L), reduced growth (R) and normal genes (N) in the combination. (C) The 10 combinations in (A) are pooled into two categories: combinations where the number of genes with normal single mutant fitness is less than 2 and combinations where the number is greater than or equal to 2. (D) Violin plot showing the distribution of the sum LID of the proteins in the two classes. (E) Distributions of the SCL connecting the protein triplets in each class. (F) The data in E are combined into two categories: the first contains protein triplets that are connected by less than eight steps, while the other contains protein triplets connected by more than eight steps. (G) The number of sGO terms shared by proteins in the triplets in each class. A triplet is deemed to share an sGO term if at least two genes in the triplet are associated with the given sGO term. Asterisks correspond to the significance of Chi-squared test comparing the negative (magenta) and neutral (cyan) feature distributions. (**H**) The percentage of gene triplets encoding proteins in the same molecular complex (Within) or from different molecular complexes (Across) that cause negative GI (magenta) or neutral GI (cyan). (**I**–**L**) feature distributions of gene triplets encoding proteins in the same molecular complex (Within, magenta color) or different molecular complexes (Across, cyan color). (I) Triplets containing at most one normal gene (*N* < 2) or at least two normal genes (*N* ≥ 2). (J) Violin plot showing the distribution of the sum LID of the proteins encoded by gene triplets in the two categories. (K) Percentage of triplets connected by a circuit of less than eight steps (<8) or at least eight steps (≥8). (L) Percentage of triplets sharing 0, 1, 2, 3, 4 and 5 sGO terms (A color version of this figure appears in the online version of this article.)

**Fig. 4.**
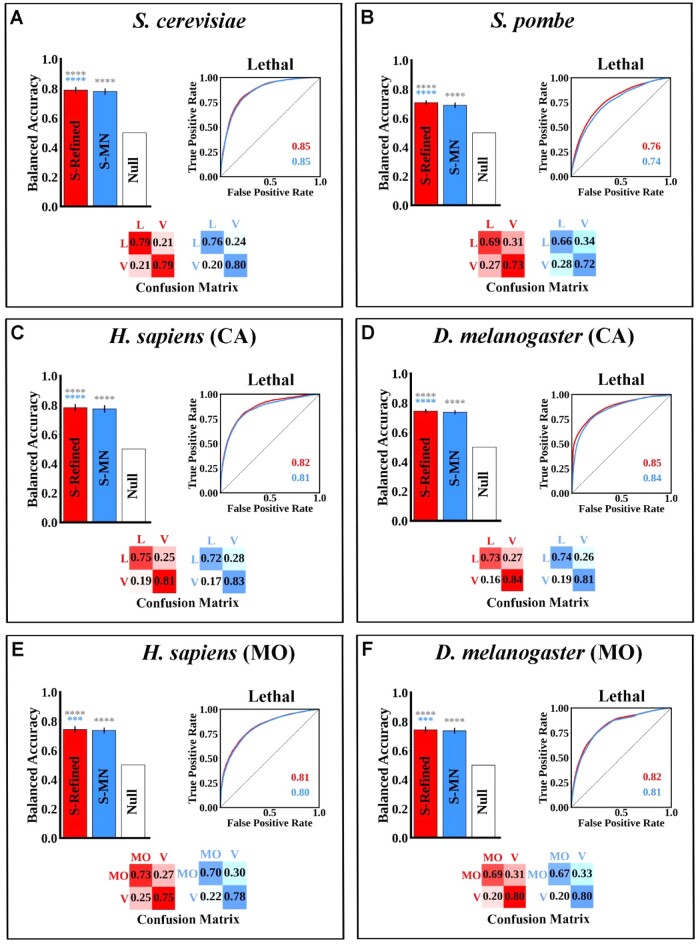
Performance of binary S-Refined and S-MN models when predicting single gene knockouts with CA or MO lethality in (**A**) S. cerevisiae, (**B**) S. pombe, (**C**) H. sapiens (cellular autonomous), (**D**) D. melanogaster (cellular autonomous), (**E**) H. sapiens (multiorganismal), and (**F**) D. melanogaster (multiorganismal). Statistical analysis (error bars and asterisks) is similar to Figure 1 with the asterisk color reflecting the model being compared

The triple gene GI full model (T-Full), which utilizes all input features, reliably outperformed the control null baseline model (Fig. 3, solid purple) on the development set. Feature selection showed that the most important prediction features are sGO terms, single mutant fitness of individual genes, LID and shortest path length (full feature selection results are reported in Supplementary Table S7). Indeed, results show that a triple gene GI refined (T-Refined) model based on these features alone performs almost as well as the T-Full model (Fig. 4, solid red). Analyses of these input features confirm this as they show reliable statistical differences between the neutral and negative classes (Fig. 3B–G).

Similar to what is observed in the double-gene GI data, within-complex triplets are more enriched in GIs than across-complex triplets. An observation that was confirmed by analyzing same set of curated protein complexes used in the double-gene knockout GI analysis (Fig. 3H–L).

Based on the T-Refined model, a simpler triple gene GI MN model (T-MN), which uses the same features, was constructed. The model has one equation specifying the log-odds of the probability of the negative class relative to the neutral class:

(5) $$\text{log}\;\left(\frac{p_{-}}{p_{n}}\right)=\omega_{0}+\omega_{1}\left(LID^{A}+LID^{B}+LID^{C}\right)+\omega_{2}SCL+\omega_{3}LLL+\omega_{4}LLR+\omega_{5}LLN+\omega_{6}LRR+\omega_{7}LRN+\omega_{8}RRN+\omega_{9}RNN+\omega_{10}RRR+\omega_{11}NNN+\omega_{12}NNL+\sum_{i}\omega_{\left(i+12\right)}\left(sGO_{i}^{A}+sGO_{i}^{B}+sGO_{i}^{C}\right)$$

The input features are guided by the above-mentioned feature analysis: the sum of LID scores, all possible unique combinations of single-mutant fitness readings of the three genes (a total of 10 combinations), the shortest circuit length (SCL) and the number of genes sharing each sGO term. Similarly to the weights in the previous equations, the coefficients $\omega_{k}$ are free parameters learned via stochastic gradient descent. Figure 3 shows that the T-MN model slightly lags behind the T-Refined model in terms of overall BA on the development dataset (Fig. 3, blue). For a full list of the T-MN classifier coefficient values, please see Supplementary Table S8.

Finally, on the withheld test dataset, the T-Full, T-Refined and T-MN models achieved very similar prediction performance, indicating that our methodology did not produce overfitting (Fig. 3 hatched purple, red and blue bars, respectively). Again, BA of 0.65–0.70 shows that the models can distinguish neutral and negative GIs despite the former class having significantly more examples than the latter (about 72 000 neutral versus approximately 3000 negative GIs).

### 2.4 The S-MN model has utility in other organisms

Next, we examined the ability of both the S-Refined and S-MN models to predict the impact of single gene disruptions on the fitness of other organisms: specifically, fission yeast, fruit flies and humans. However, the available datasets for these organisms differ from those available for budding yeast in two important aspects. First, the volume of bioinformatic data is not as extensive as the ones available for budding yeast, making it challenging to construct an S-Full black box model equivalent to the one generated for budding yeast. Second, even though the effect of knocking out a given gene on cell viability, i.e. its cell-autonomous (CA) role, can be directly evaluated in unicellular organisms such as fission yeast, the same cannot be said for fruit flies and humans, as those organisms likely possess sets of genes that are essential only at the multicellular level, i.e. multi-cellular organismal (MO) lethal.

To identify cell autonomous CA genes, we obtained data from single mutant gene knockouts in fission yeast (Kim *et al.*, 2010; Ryan *et al.*, 2012), the fruit fly S2 cell line (Viswanatha *et al.*, 2019) and the human embryonic stem cell line HUES62 (Shalem *et al.*, 2014). Given that the data for the latter two cell lines were collected from studies that utilized CRISPR, a method that is more specific than RNAi and that cell viability was directly evaluated, we can confidently state that the reported results for these target genes are CA-specific.

Since no model development was performed on these datasets, we evaluated models using the input feature types identified in the budding yeast dataset. In all three organisms, the S-Refined and S-MN models were significantly better than the null model, with confusion matrices showing a strong performance in predicting both the lethal and normal classes (Supplementary Fig. S7A–C). However, only in the human datasets do we observe a moderate ability of the S-MN model in distinguishing among genes that cause reduced growth when knocked out from other genes that cause lethality or normal growth. For a full list of the three-way organism-specific S-MN model coefficient values, see Supplementary Table S3, sheet 1.

The tremendous interest in identifying essential/lethal genes in specific pathological cell contexts (Cheng *et al.*, 2014; Li *et al.*, 2012; Luo and Wu, 2015) prompted us to reevaluate our model’s performance when the output classes of single mutant fitness are grouped into only two classes: viable (including reduced growth and normal) and lethal. Both the S-Refined and S-MN models perform significantly better when this grouping scheme is utilized (Fig. 4A–D). The BA score of about 0.77 in the human and fruit fly datasets is especially impressive given the significant class imbalance in those organisms (16 000 viable human genes versus 586 lethal and 12 000 viable fruit fly genes versus 1214 lethal). Again, this BA score indicates that the models are not trivially predicting the majority class all the time. For a full list of coefficient values for the binary organism-specific S-MN models, see Supplementary Table S3, sheet 2.

Recently, a human sequencing dataset has become available in which polymorphisms that cause early stop codons in various human subject genes were detected (Karczewski *et al.*, 2020). Using this data, we extracted a list of genes that are essential for multi-organismal (MO) viability and those that are not by subtracting CA lethal genes identified by Shalem and others (Shalem *et al.*, 2014). We also used a similar approach for fruit fly datasets by subtracting CA lethal genes identified by Viswanatha *et al.* (2018) from the list of lethal genes deposited in Flybase (Thurmond *et al.*, 2019; For the methodology used to identify MO genes see Supplementary Material and Methods). Both the S-Refined and S-MN models perform well in distinguishing MO lethal from MO viable genes (Fig. 4E and F).

This performance prompted the next question: can our models distinguish between CA, MO and viable genes? Results show that the S-Refined and S-MN models can indeed distinguish between these three classes in humans and fruit flies (Supplementary Fig. S8). We note that on the fruit flies, the viable class is 10 and 5 times as large as the CA and MO classes, respectively, yet the model still achieves BA > 0.6, showing that it is not merely predicting the viable class all the time.

### 2.5 D-Mn models can predict GIs in other organisms

In fission yeast, a methodology that can generate extensive double mutant pairs between non-essential genes has been used to produce a comprehensive list of GIs (Roguev *et al.*, 2007; Roguev *et al.*, 2018). We used this list in combination with data from BioGRID to create a hybrid dataset. The D-Refined and D-MN models perform reasonably well and can distinguish most GI categories, with the exception of the positive GI class (Supplementary Fig. S5B).

**Fig. 5.**
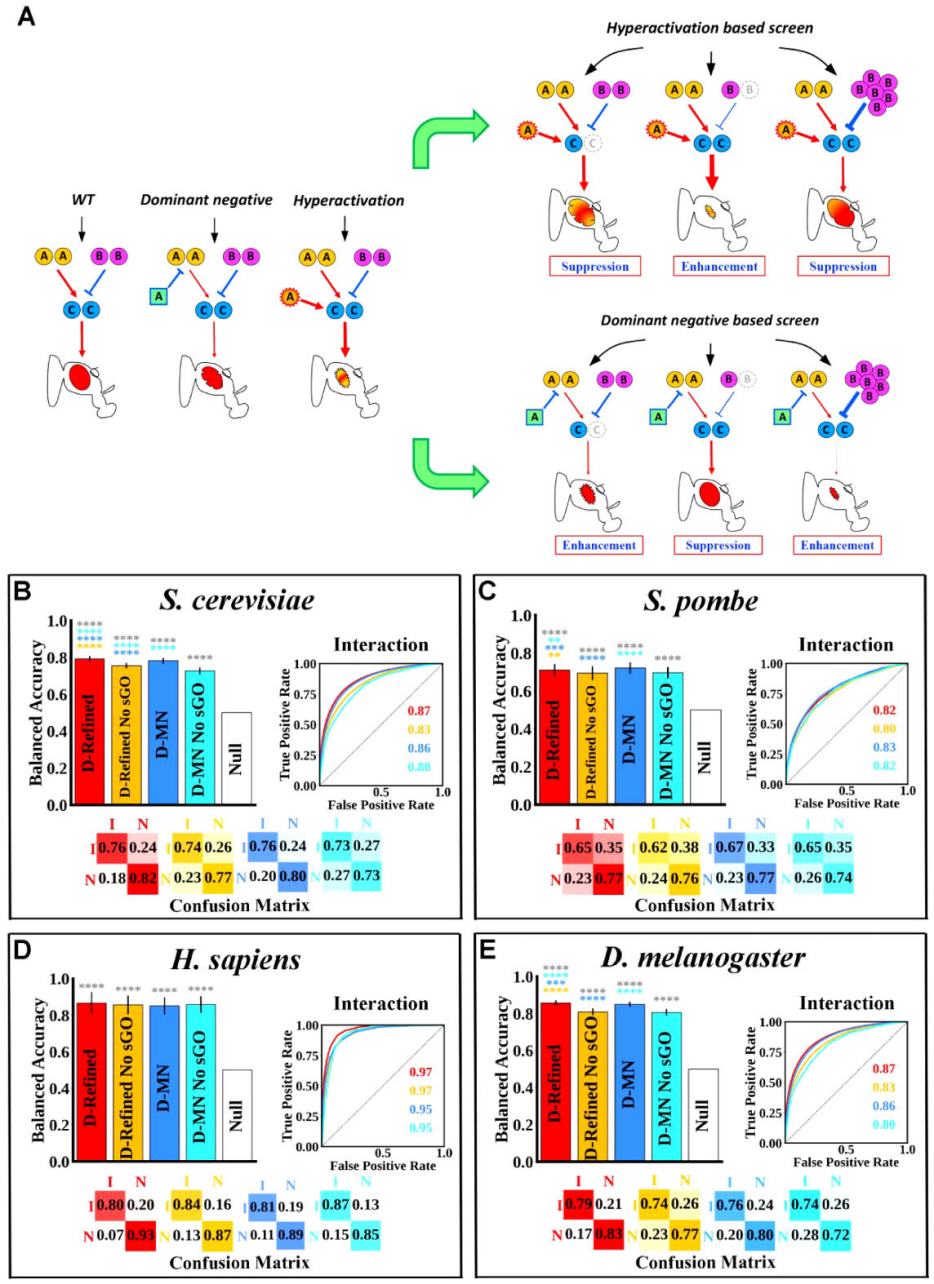
Performance of the D-Refined and D-MN models when predicting GIs in *S. cerevisiae*, *S. pombe*, *H. sapiens* and *D. melanogaster*. (**A**) Representation of schemes used to generate GIs in a hypothetical signaling pathway controlling fruit fly eye development. Gene A is in a positive signaling relationship with gene C (red arrow), gene B is in an inhibitory relationship with gene C (blue line) and gene C sends a signal that promotes eye development. In this example, a transgene is used to express either dominant negative or hyperactive forms of gene A specifically in the eye: the dominant negative gene A reduces pathway signaling which produces mildly rough eyes, while the hyperactive form causes a reduced eye with abnormal pigmentation. Both transgenes can be used either in a haploinsufficiency screen in which one copy of gene B or C is removed (gray circles), or overexpression screen in which gene B is overexpressed. Thickness of arrows and lines reflects changes in signaling levels. (**B**–**E**) Performance of the D-Refined (red), D-Refined with no sGO (orange), D-MN (blue), D-MN with no sGO (cyan) and null GI model (white) when predicting interacting (I) versus neutral (N) on the *S. cerevisiae* (B), *S. pombe* (C), *H. sapiens* (D) and *D. melanogaster* (E) GI datasets. Statistical analysis (error bars and asterisks) is similar to Figure 1A with the asterisk color reflecting the model being compared (A color version of this figure appears in the online version of this article.)

Unlike budding and fission yeast, most human and fruit fly GI datasets were generated using either enhancement or suppression of an overexpressed hyperactive or dominant negative target gene (Grimm, 2004; Jorgensen and Mango, 2002; St Johnston, 2002). This is typically followed by introduction of gene manipulation strategies that include RNAi, haploinsufficiency (removal of one copy of a gene in a diploid genome) or systematic overexpression of target genes (Belfiori-Carrasco *et al.*, 2017; Boutros and Ahringer, 2008; Gregory *et al.*, 2007; Raymond *et al.*, 2004; Therrien *et al.*, 2000). These different manipulations can potentially convert what is classified as a negative GI in certain experimental contexts into a suppressor GI in other contexts (Fig. 5A). While these differences can be addressed by systematically identifying the experimental approach used within each primary reference cited by BioGRID or FlyBase, manual revision of existing curated entries is a daunting task. Therefore, to reduce this context-dependent nature of observations, we grouped negative, positive and suppression observations in all organisms into a single category simply referred to as GI. Another disadvantage of the BioGRID and FlyBase databases is their lack of reporting on non-interacting gene pair data that can be placed in the neutral class. To overcome this lability, we randomly generated such a class by negative sampling such that no neutral pair was ever present in the GI pairs dataset, an approach that has been done previously (Benstead-Hume *et al.*, 2019).

In all tested organisms, both the D-Refined and D-MN models exhibit superior performance compared to the null model (Fig. 5B–E; for a full list of coefficient values and confidence intervals for the binary classification tasks, see Supplementary Table S6, sheet 2). We note that the BA of the D-Refined and D-MN models are highest on the human and fruit fly GI datasets, which is most likely due to the randomly sampled neutral GIs in those organisms. However, even though these two datasets where explicitly constructed to have a high negative to positive ratio (1 000 000 neutral GI versus a few thousand GIs) to mimic the imbalance in the yeast GI dataset, the high BA of D-Refined and D-MN models shows that they perform well on *both* neutral and GI classes and that they are not trivially predicting the majority class, i.e. they are not predicting neutral GI all the time.

Our previous analyses in budding yeast indicate that most GIs occur between gene pairs that encode units of the same molecular complexes or pathways. Therefore, it is possible that both the D-Refined and D-MN models will only show a minor drop in performance if input features are limited to those that are good proxies for complex/pathway membership and the impact of each gene in the pair on viability, namely LID, shortest path length and single mutant fitness. To examine this possibility, models without sGO (D-Refined-No sGO and D-MN-No sGO) were evaluated on the binary classification task (interacting versus neutral). With the exception of humans, which show no difference in performance, all other GI predicting models that are missing the sGO components show a modest, but statistically significant, drop in performance (Fig. 5B–E, cyan). This indicates that the models’ GI predictions are not simply based on common biological function between the target gene pair, as might be indicated by them sharing sGO terms that reflect biological function. Nevertheless, the observed modest and statistically significant drop in performance when these sGO terms are removed justifies their inclusion as an input feature in both the D-Refined and D-MN models.

### 2.6 Mn models show superior performance to previously published models

Given the existence of previous computational models designed to predict the impact of single gene knockout on organismal viability, and the development of models that can predict negative GIs, it is critical to compare the construction/architecture and performance of the MN models described here to these previously published models. However, such direct comparisons pose some challenges: First, the same performance metrics must be used for the comparisons to be valid. Unfortunately, most of the previously published models employ AUC-ROC (Al-Aamri *et al.*, 2019; Benstead-Hume *et al.*, 2017; Gabriel del Rio, 2009; Mistry *et al.*, 2017; Wong *et al.*, 2004; Wu *et al.*, 2014) to assess model performance, which is not an appropriate metric for imbalanced datasets (Davis and Goadrich, 2006; Saito and Rehmsmeier, 2015). Second, the majority of single mutant fitness models were not evaluated in a training/testing setup since they make very strong and restrictive assumptions about the relationship between input features and output phenotype (i.e. gene X is essential because it has a high score in only one or two features; Gabriel del Rio, 2009; Luo and Qi, 2015; Luo and Wu, 2015; Mistry *et al.*, 2017). Third, some of the GI models were evaluated on artificially balanced test sets, which can inflate their performance (Benstead-Hume *et al.*, 2019; Campos *et al.*, 2019; Wu *et al.*, 2014). Fourth, the majority of previous models only predict binary outcomes (i.e. lethal/essential genes versus everything else or negative GIs versus everything else) while the MN models have a harder task of predicting more outcomes (lethal, reduced growth and normal for the single gene knockout prediction, and negative, neutral, positive and suppression for GI prediction). Nevertheless, some measure of comparison is needed, and the budding yeast dataset was selected for this purpose as it is the most widely used.

Published models that are recent and/or have high reported performance were selected for the comparisons, and all were evaluated via the exact same pipeline used to evaluate the MN models described in this work (the same cross-validation splits and evaluation metrics of BA, confusion matrix and AUC-ROC) without artificial balancing. In all cases, these models were compared to the interpretable binary S-MN models that predicts lethal versus viable, and the binary D-MN models that predict negative versus everything else (for details, see the Comparisons with Other Models Section in the Supplementary Materials and Methods).

For the single mutant fitness, we compared the models developed by Campos *et al.* (2019), Mistry *et al.* (2017) and Luo and Qi (2015). The Campos *et al.* model uses 9920 amino acid sequence features of proteins encoded by the budding yeast genome to distinguish essential genes. The Mistry *et al.* model utilizes the DiffSLC graph centrality metric developed by the authors to distinguish essential genes. This centrality uses a weighted combination of eigenvector centrality and the sum of protein co-expression correlations of a particular protein to characterize the importance of the gene that encodes it. Finally, Lou *et al.* developed the LID-IDC metric, which uses a weighted combination of LID centrality and complex membership of a protein to determine the importance of the gene that encodes it. The single gene S-MN model outperforms all the above-stated models in overall BA, confusion matrix and AUC-ROC (Supplementary Fig. S9A).

Turning to the GI tasks, the models developed by Benstead-Hume *et al.* (2019), Yu *et al.* (2016) and Alanis-Lobato *et al.* (2013) were selected for comparison. The Benstead-Hume *et al.* model exploits conserved patterns in the protein interaction network topology both within and across species to predict negative GIs. The model proposed by Yu *et al* utilizes the hierarchical organization of the full set of budding yeast GO terms for its predictions. Finally, the model proposed by Alanis-Lobato *et al.* uses topology-based pairwise Adjusted-Czekanowski–Dice Dissimilarity (ACDD) feature for negative GI prediction. The D-MN model outperforms all the above-stated models in overall BA, confusion matrix and AUC-ROC (Supplementary Fig. S9B). Note that while the models by Yu *et al.* (2016) and Alanis-Lobato *et al.* (2013) achieve an AUC-ROC of 0.74 and 0.67, respectively, their confusion matrices illustrate a clear problem. Yu *et al.* (2016) model only identifies 47% of the negative class, with the remaining 53% being incorrectly predicted as not negative, while the Alanis-Lobato *et al.* (2013) model does the opposite: it only identifies 41% of the non-negative class and incorrectly predicts the remaining 59% as negative. Of course, using a different decision threshold to generate the confusion matrices might ameliorate the issue for both models, but we can say that at the common decision threshold of 0.5, both models confound the two GI classes. The discrepancy between AUC-ROC and confusion matrices shows why it is important to use multiple evaluation metrics, including those that can handle class imbalance.

In conclusion, the analysis described above demonstrates that the MN models frequently perform better than previously published models when evaluated on equal grounds. Also, as stated before, unlike the majority of these models, the MN models offer direct interpretability of the relationship between input features and outputs, which is crucial for understanding the basis for a model’s predictions.

## 3 Discussion

In this article, we report the development of MN models that can predict the impact of genetic disruptions on organismal viability. These models were developed via a sequential process that initially started with more than 300 gene input features classified into seven broad categories which were then pared down via feature selection. The selected features were then used to develop equally-predictive open box MN models that provide clear explanations of the form: if the value of feature X increases by one unit, the odds of predicted lethality by the S-MN or GI by the D-MN models increase by factor Y.

The S-MN model, which can predict the impact of single gene knockouts on organismal viability, is composed of three input features: a set of sGO terms that broadly describe the biological process, molecular function and the cellular compartment of the target gene product, the LID score, which measures the connectivity of the molecular complex to which the protein encoded by the target gene belongs, and finally the level of homology between the target gene and other genes encoded by the host genome. The D-MN model predicts GIs and utilizes the sGO terms, LID score, single-mutant fitness of both genes in a given gene pair, and the distance between the target gene pair’s protein products in the PPI network, features that are clearly related to whether the target gene pairs encode proteins in the same molecular complex or pathway. A third MN model, T-MN, which uses the same features as D-MN, also achieves good performance in predicting negative GIs in triple gene knockout in budding yeast.

It is worth noting that, when compared on equal grounds, both S-MN and D-MN models outperform previously published models. Furthermore, our analyses show that the D-MN is not simply predicting GIs involving gene pairs with direct protein-protein interaction or common biological function as reflected by sGO terms (See Supplementary Materials and Methods and Supplementary Fig. S10).

The input features used by these models give relatively straightforward explanation of their abilities to predict the impact of genetic disruption on organismal fitness: The more the target gene participates in essential molecular processes, as described by sGO terms, the more likely its removal will reduce cell viability. This impact will be amplified if the target gene encodes a protein that is part of a highly connected complex, as reflected by its LID score, but it gets reduced when the genome contains other genes with enough sequence similarity to function as backups. The further removal of another target gene in such a background can modify its impact on organismal viability if both genes encode proteins that are part of the same complex or pathway, as indicated by the shortest path length. Even though the S-MN, D-MN and T-MN models differ in the types of input features used, they do share some features such as LID centrality and sGO terms but their contribution is task-specific (see Supplementary Materials and Methods and Supplementary Fig. S11).

The input features utilized in all three models can easily be curated in other organisms. Indeed, when the S-MN and D-MN models were trained only on the budding yeast dataset, they were still able to perform well on other organisms’ datasets (without training on those organisms). For details, please see Supplementary Materials and Methods and Supplementary Figure S12.

In all cases, even though the MN models are only composed of first-order terms, their performance still matches or is close to the NN models, indicating that interactions among different input features within a given MN model do not contribute synergistically to prediction performance, with each feature contributing additively and independently to the final predictions.

It is worth stating that these MN models have important biomedical applications, such as the identification of disease-specific essential genes (Benstead-Hume *et al.*, 2017). Due to these potential biomedical applications, we have created a website (see Availability and Implementation) that provides a list of novel GI predictions at different thresholds for all tested organisms (Supplementary Fig. S13). These predictions can be used by researchers to obtain generalizable insights in order to design large-scale genetic experiments in a rational, targeted and economically feasible way.

## Supplementary Material

btac519_Supplementary_Data
