# Supplementary material for: Predicting and explaining the impact of genetic disruptions and interactions on organismal viability
Source: Bioinformatics. 2022 Jul 21;38(17):4088–99. doi: 10.1093/bioinformatics/btac519 (PMC9438956; doi:10.1093/bioinformatics/btac519)
Supplement: btac519_Supplementary_Data [file btac519_supplementary_data.zip › btac519_Supplementary_Data/Supplementary Material and Methods copy.docx]

**Supplementary Materials and Methods**

**Data and Code Availability**

All code used to generate results and plot figures in this manuscript is available at our Github repository at <https://github.com/KISRDevelopment/cell_viability_paper>. The repository also contains a link to the GI prediction website.

**Supplementary Analyses**

**The D-MN Model is not just predicting direct Protein-Protein interactions**

Even though the D-MN model outperforms most of the double gene GI models (shown later), it could simply be predicting direct binary interactions between proteins encoded by the target gene pair. This concern is justified because the D-MN model utilizes features derived from the coprecipitation network (LID and shortest path length) and includes the sGO terms *kinase* and *phosphatase* that are responsible for the direct phosphorylation/dephosphorylation connection, and the term *transcription binding* that can reflect one gene encoding a transcription factor that directly binds to the DNA of the other (Supplementary Figure 10A).

To test this hypothesis, we evaluated a binary version of the D-MN model on the task of predicting binary coprecipitation, phosphorylation/dephosphorylation, and transcription factor binding relationships in budding yeast (Supplementary Figure 9B). The D-MN model was not able to predict transcription factor binding, but it did perform well in predicting coprecipitation and phosphorylation. The latter result is not surprising since the model uses input features that directly correspond to protein-protein binding, i.e., shortest path length and *phosphorylation/dephosphorylation* sGO terms, making it easier for the model to predict those relationships (Supplementary Figure 10C, solid bars). However, to be thorough, we evaluated a variant of the D-MN model that lacks all coprecipitation topology features and the above-stated sGO terms (Supplementary Figure 10C, hatch bars). The modified D-MN model exhibits only a minor drop in GI-prediction performance and does not predict the above-stated direct protein-protein relationships. In addition, as shown previously (Figure 6B-D, cyan), D-MN models missing all sGO terms, a feature that can indicate a common biological function, only exhibit a minor, but statistically significant, drop in performance as compared to full D-MN models, indicating that the models’ GI predictions are not simply based on common biological function. The above analysis indicates that the GI predictions generated by the D-MN model are not trivially linked to direct binary protein-protein interaction or to genes with a common biological function.

**The shared input features of the MN models have task-specific contribution**

Even though the S-MN, D-MN, and T-MN models differ in the types of input features used, they share features such as LID centrality and sGO terms. These features passed the feature selection procedure but the levels of their contributions might be task-specific.

To examine this issue, these input features were removed one-at-a-time in S-MN, D-MN, and T-MN models and the resulting cross-validation performance was evaluated. The percentage drop in overall balanced accuracy of these modified models relative to the regular MN models should serve as a good indicator of their importance (Supplementary Figure 11). For the S-MN model, the most important feature is the sGO (12.5% drop in performance upon removal), which can be explained by the fact that the sGO terms are a better proxy for gene essentiality than location of the protein they encode in the PPI network (Supplementary Figure 11A). Indeed, the location of a protein within a PPI network might affect its efficiency in carrying out its role, but it will not change its molecular function as described by its sGO terms. For example, the basic molecular function of RNA polymerase is dependent on its amino acid sequence, and it will not adopt the identity of a cytoskeletal protein if its location within the PPI network is altered.

Additionally, upon examining the effect of removing the top 20%, 40%, 60%, and 80% of sGO terms enriched in the lethal genes class, a reduction of model balance accuracy to less than 58% is only observed when more than 80% of lethal gene enriched sGO terms are removed (data not shown).

For the D-MN model, the sGO terms’ influence is reduced to 7.5% with the most important feature being the single mutant fitness (SMF) (16% drop in performance; (Supplementary Figure 11B). The drop in the importance of the sGO terms in the D-MN model is most likely due to the fact that SMF encompasses some of the contributions of sGO terms, percent identity, and LID to organismal viability. Finally, unlike the D-MN model, the SMF input feature is less important in the T-MN model (with only 2.5% drop in performance; Supplementary Figure 11C). This might be due to the fact that the number of lethal genes in the trigenic interaction dataset is much less than that in the double gene knockout dataset.

It also worth noting that in the case of the shared sGO terms, their coefficient values do differ from one model to the other. For example, the sGO term “mitochondrion organization” in budding yeast has coefficient values of 0.135, -0.0145, and -0.301 in the binary versions of the S-MN, D-MN, and T-MN models, respectively.

All of above analysis shows that the contribution of the shared input features is task-specific. However, the presence of those shared “universal” features could be considered a strength rather than a liability, reflecting the fact that these models can predict the impact of genetic disruption (whether single, double, or triple) on the viability of the cells, an open system in which the impact of given gene on overall viability cannot be completely segregated from other genetic disruptions occurring within the genome.

**The MN models have partial universality**

Even though the MN models use sufficiently informative and similar input features to predict phenotypes in all four organisms tested, the contribution of these input features might be organism-specific. This issue can be addressed by training both the S-MN and D-MN models only on the budding yeast dataset and then testing directly on the single and double gene disruption outcomes in another organism.

The budding yeast-trained S-MN model performs better than the null model in fission yeast and humans, but not in fruit flies (Supplementary Figure 12 A-D, blue). Taken together, these results suggest the presence of only a moderate degree of universality in the budding yeast-trained S-MN model’s ability to predict essential genes in different organisms.

The D-MN model trained on only the budding yeast data was able generalize extremally well on fission yeast, humans, and fruit flies (Supplementary Figure 12E-G, blue). One possible explanation for ability of the D-MN but not the S-MN is that the sGO terms, which are more influential in the S-MN, are not the ones responsible for the observed generalization, but rather it is the features that are proxies for complex/pathway membership (i.e., LID and shortest path length) and single gene viability. This is supported by the observations that D-MN models without sGO terms still perform well relative to those D-MN models without sGO terms (Figure 6 B-E, cyan), and that the budding yeast D-MN model without sGO terms generalized very well in other organisms (Supplementary Figure 12E-G, cyan). These observations agree with idea that most GIs occur between gene pairs encoding units of the same molecular complex or pathway, which tend to have evolutionarily conserved signaling relationships ^55,56^.

**Supplementary Methods**

**Modeling**

***Neural Network Models***

To construct initial black box models, we used standard feed-forward NNs. The NNs are composed of an interconnected group of units called artificial neurons, which mimic the function of neuronal cells, organized in a hierarchy of connected layers ^1^. In the first layer, a group of units called input neurons receive input features. The input neurons are connected to neurons in the hidden layer where each hidden neuron computes a weighted summation of input features and then applies a non-linear transformation to that summation. The learning procedure for a NN involves tuning the weights between each layer such that the output of the network is as close as possible to the observed outputs in the dataset. This is done via a procedure called backpropagation which incrementally changes weights in the network, starting at the last layer and going backwards.

Given the heterogeneity of our feature sets (e.g., topology, sGO terms, redundancy, etc.), we created a custom NN architecture that explicitly embodies this feature set heterogeneity into the NN. Each feature set is connected to a sub-NN with one layer of 5 tanh units (tanh is known as the hyperbolic tangent activation function and it maps values in the range -∞ to +∞ to values in the range of -1 to +1) and the outputs of those sub-NNs are then concatenated together and passed through a hidden layer of 5 tanh units that is wired to the output of the neural network. For localization features, we used a distinct sub-NN architecture because localization features are two dimensional; they measure target protein expression levels in different cellular compartments across time. Here, the same sub-NN with one layer of 5 tanh units is applied to the time series of protein expression levels at each cellular location. In total, 16 sub-NN compartments were constructed generating an output of $16\times5=80$ units which are connected to a layer of 5 units. For the output layer, we use a *softmax* layer^2,3^ with 3 units (corresponding to lethal, reduced growth, and normal). The *softmax* layer takes a set of continuous inputs (which can be negative or positive) and transforms them into a set of 3 positive numbers that sum to 1.0 and which represent the predicted probabilities of the corresponding classes. For example, if the actual observation is reduced growth, the network should assign a high probability to the reduced growth bin and low probabilities to the other two bins. In the case of binary classification, the *softmax* layer outputs 2 units. For the architecture diagram, see Supplementary Figure 2A.

For double-knockout GI-predicting functionality, the NN architecture was modified to account for the symmetry between genes in a pair (i.e., pair A-B is the same as B-A). Specifically, the model is given explicit knowledge that the features belong to two genes. Siamese architecture ^4^ composed of three modules was used: Two identical sub-NN modules that transform the feature sets of each gene in the pair. The above stated sub-NN has an identical architecture to the one used in the case of single mutant fitness datasets (that is, it accepts the feature sets of a gene and outputs a vector of 5 units). The third module containing a separate sub-NN transforms to process the pairwise feature set that jointly characterizes both genes in the pair. It accepts all pairwise feature sets and also outputs a vector of 5 units. To summarize, the feature sets of gene A and B and the set of pairwise features are transformed into three vectors of 5 units each. Those are concatenated together and connected to the final output softmax layer with either 4 units (negative, neutral, positive, and suppression) or 2 units (interacting and neutral). For the architecture diagram, see Supplementary Figure 2B.

For triple knockout GI-predicting NNs, the above-mentioned architecture is modified in two ways: First, a tri-head Siamese architecture is used. This architecture is composed of six sub-NN modules which transform the feature sets of each gene in the triplet using identical sub-NN. Second, instead of having one pairwise feature, the triple GI NN accepts three pairwise input feature sets, one for each gene pair in the triplet (e.g., for triplet ABC, there are three pairs: AB, AC, and BC each with its own sub-NN modules). These features are fed into another tri-head Siamese architecture which transforms the pairwise features of each gene pair in the triplet using an identical sub-NN. In summary, the input feature sets are transformed into six vectors containing 5 elements each (three vectors for the three genes, and three vectors for the three pairs of genes). Those vectors are combined by summing the first three vectors together and the last three vectors together and concatenating the resulting two 5-element vectors. The result is then connected to the final softmax layer with 2 units (negative versus neutral). For the NN architecture diagram, see supplementary Figure 1C.

With the exception of the null model, all models are trained via stochastic gradient descent, and were implemented in the Tensorflow 2 and Keras Python libraries, which allow developers to implement regular NNs as well as custom computational architectures.

***MN Models***

The weights or free parameters of the MN models are simply the coefficients in the equations presented in the main text which are learned via stochastic gradient descent. As stated earlier, both models are implemented in the Tensorflow 2 and Keras libraries, and are thus treated the same way as the regular NN models.

***Null Model***

The null model simply predicts the average class distribution over the training set. That is, it predicts the same output probabilities, regardless of the input features.

***Training Objective***

To train a model, an objective function is needed (also known as the loss function). This function quantifies the quality of a particular set of free parameters of the model. If a set of free parameters of the model leads to predictions that are close to the observations, the objective function would assign a lower value (smaller loss) than a set of parameters that leads to predictions that diverge from the observations (higher loss). A typical loss function used by models that predict categorical outcomes (e.g., lethal versus viable, negative versus neutral versus positive, etc.) is the categorical cross-entropy function. This objective function attempts to maximize the probability of the training observations and it is defined as:

$$\mathcal{L}_{CE}=-\frac{1}{N}\sum_{i}^{N} \sum_{j}^{M} y_{ij}log(p_{ij})$$

Where $N$ is the total number of observations, $M$ is the number of output classes, $y_{ij}$ is 1 if the output class of the *i*th observation is *j*, and $p_{ij}$ is the model’s predicted probability that the output class of the *i*th observation is *j*.

One particular challenge with using the standard cross-entropy objective function in biological data is the high degree in imbalance among output classes. In our case this is especially pronounced in the GI prediction task, with most observations falling in the neutral class. This imbalance is problematic because the training procedure might generate a model that simply predicts the majority class all the time. To correct for this, a specialized training objective function known as the weighted cross-entropy loss is used. This function forces the training procedure to pay equal attention to all output classes, regardless of how many observations they contain. This loss eliminates the need to explicitly balance out the training set by under- or over-sampling observations, which is advantageous because under-sampling removes valuable observations, while over-sampling increases training time. Mathematically, the weighted cross-entropy objective is defined as:

$$\mathcal{L}_{WCE}=-\frac{1}{N}\sum_{i}^{N} \sum_{j}^{M} {\frac{1}{C_{j}}y}_{ij}log(p_{ij})$$

where $C_{j}$ is the number of observations of the j^th^ class in the training set. In other words, the weighted cross entropy function sums the average cross-entropy in each class, thereby treating all classes equally regardless of their size (e.g., a dominating class would have a large $C_{i}$, reducing the penalty on the model when it mispredicts members of that class, and vice-versa with small classes).

***Training Procedure***

Given the weighted cross-entropy objective, NNs are trained with an algorithm known as backpropagation in which a forward pass through the network computes the activations of all neurons, followed by a backward pass from the output to the inputs to update the weights of the network. Standard backpropagation has a learning rate parameter which quantifies how big of an update to perform on the weights. Larger learning rates allow the model to make rapid changes to the weights but may lead it to potentially “over-shoot” optimal solutions, while smaller weights lead to slower learning. The weight update is performed after a full run over the training data, known as an “epoch” of training. The network is trained for a pre-specified number of epochs and the weight parameters at the final epoch are used. However, standard backpropagation can suffer from slow learning and convergence, so techniques have been developed to address this problem. First, mini-batch training was used in which weight updates are performed more frequently (after small batches of training data). In the single gene disruption datasets, a mini-batch size of 10,000 observations for NN models and a mini-batch size of 100 observation for the MN models were used. In double gene disruption datasets, a mini-batch size of 10,000 observations for NN and MN models were used. Second, the NADAM ^5^ optimizer, which uses adaptive learning rates and momentum to accelerate convergence and learning, was used. The defaults parameters of the NADAM optimizer in the Keras Python module were used: 0.002 learning rate, first moment exponential decay rate $\beta_{1}=0.9$, second moment exponential decay rate $\beta_{2}=0.999$, and schedule decay of $0.004$.

***Preventing Overfitting***

NN models are prone to the problem of overfitting: a situation where the model learns spurious noisy patterns in the data, reducing its ability to make accurate predictions on new inputs ^6^.

Visually, one can see when the model overfits by looking at the plot of training and testing loss versus the number of epochs. At the start of training, both losses would decrease, but when overfitting happens the testing loss would increase while the training loss would continue to decrease. Ideally, one would pick the epoch before the testing loss starts to increase as the point to stop training the NN. In early stopping, a portion of the training dataset is withheld from the model for validation purposes. During training, the model’s performance on the validation set is monitored after every epoch and training stops when the model’s performance on the validation set starts to degrade. At this point, the model’s learned weights are saved and used as the final fitted parameter values. An early stopping has a parameter known as the “patience”, which is how long the training procedure will wait before stopping due to no improvement in the loss function. In single gene disruption datasets, the patience parameter was set to 50 epochs, so the training will stop if there are 50 consecutive epochs in which no improvement in loss has been made. In double gene disruption datasets, the patience was set to 10 epochs. When training stops, the weights associated with the epoch with the lowest validation loss are picked as the final fitted weight values.

***Evaluation Metrics***

To mitigate class imbalance, weighted-categorical cross entropy loss function was used during training. This approach downweighs frequent classes to prevent them from dominating classification performance. We also use performance metrics that are appropriate for unbalanced datasets, such as Balanced Accuracy (BA), which calculates the average of the per-class accuracies ^7,8^, and confusion matrix, which shows a complete picture of classifier performance at a given decision threshold by counting the frequency of all predicted versus observed class combinations. To compute balanced accuracy and the confusion matrix, we quantize the soft probabilistic output of our models by predicting the class with the highest output probability.

***Cross-Validation***

Cross-validation was used to evaluate a model’s test performance on a given organism’s single, double, or triple gene disruption datasets. In cross-validation, the input dataset is split randomly into *N* non-overlapping subsets and the model is trained and tested *N* times. Each time, the model is trained on *N-1* subsets and tested on the remaining subset. Test performance was evaluated according to the standard metrics used throughout this work (balanced accuracy, confusion matrix, etc.). To further reduce variability, we used 10 replications of 5-fold cross validation (80% of genes in training and 20% in testing) for single gene disruption datasets and 10 replications of 4-fold cross validation for double gene disruption datasets due to sparser output distributions (75% of genes in training and 25% in testing). In the GI datasets, the additional constraint that only new genes can be seen during test time was imposed; the model should not have seen either gene in a test pair during training. This is to prevent the model trivially using the input features to memorize the identity of the genes. In the triple GI budding yeast dataset, this constraint was removed due to the sparsity and small size of the dataset. The performance metrics are averaged across all training/testing runs (50 and 40 runs in single and double gene disruption datasets, respectively). The workflow is summarized in Figure 1C.

***Development and Testing***

Since our model development methodology in Figure 1 is multi-stage, it is imperative that the final evaluation of all models is done on data that was not involved in model development. Therefore, we split the yeast dataset into development dataset (containing 80% of the data) and a testing dataset (containing the remaining 20%). All stages of model development, including feature selection, were performed only on the development dataset using repeated cross-validation described previously.

For the double gene GI datasets, we impose the additional constraint that no overlap exists between the sets of genes involved in the two portions: meaning that all pairs involving a gene present in development set were excluded from the test set. This splitting process is known as stratification by gene and it ensures that models cannot do well by simply memorizing the identity of the genes in the training set.

Due to the limited size of triple gene knockout dataset, attempting to stratify the two portions by genes would drastically reduce the size of both portions (an average of 4,315 triplets in the development set, and 61 triplets in the testing set when genes are split in 80%-20% proportions). Thus, gene stratification was not imposed on the triple gene knockout GI dataset.

In all cases final evaluation of the selected models was performed on the test dataset. Note that for organisms other than the budding yeast, no such splitting is performed because the models’ input features have already been determined from the budding yeast dataset. Only cross-validation is performed on those organisms’ datasets.

***Hyperparameter Optimization***

There are infinitely many possible configurations of NN architectures and training settings. To ensure that the suitability of the NN models being used, we perform grid-search hyperparameter optimization to systematically evaluate all combinations of a given set of hyperparameter settings and select the optimum one.

For the S-Full model, we ran hyperparameter optimization on the *S. cerevesaie* development set by sweeping the hidden layer sizes of the input sub-NNs (one layer of 1, 5, or 10 units, or two layers of 5 and 2 units, or two layers of 10 and 5 units, respectively), pre-output embedding sizes (1, 5, or 10 units), and learning rates (0.1, 0.01, or 0.001). In total, there are $5\times3\times3=45$ possible combinations of hyperparameters. While those combinations are by no means comprehensive, we believe they encompass the most important architectural and training choices of the neural network models. For each combination, the average test set overall balanced accuracy was assessed over 10 replications of 5-fold cross-validation (Supplementary Table 1, sheet 1). Results show that the default configuration described previously (5 units in the hidden layers of the input sub-NNs, 5 units in the pre-output embedding layer, and a learning rate of 0.001) achieves the best performance. While for the D-Full model, Results show that the default configuration is only 1% behind the optimal configuration in terms overall balanced accuracy (Supplementary Table 1, sheet 2).

***All Subset Feature Selection***

Feature selection was used on the budding yeast single and double gene disruption datasets to determine which input features are important to the model’s predictive performance. All possible combinations of input feature sets were enumerated. In the case of the single gene disruption dataset, there are 7 feature sets (topology, sGO, redundancy, phosphorylation, transcription, abundance, and localization), yielding a total of 127 combinations. In the case of double and triple gene disruption, we add the single- mutant fitness and pairwise features, yielding a total of 512 possible combinations. However, we limit the search by considering phosphorylation and transcription jointly, as well as abundance and localization jointly, yielding a total of 127 combinations of input feature sets to search through.

For each input feature set, a NN model is trained and evaluated via cross-validation on the development set. The rough “refined” model that achieves the best cross-validation performance is selected based on overall balanced accuracy and use of the fewest number of features. Since some feature sets involve multiple features (e.g., topology, redundancy, pairwise features, etc), a second round of feature selection is performed to determine the exact feature within a feature set that contributes the most to predictive performance. Here, all features in a feature set are examined one at a time, while the other feature sets in the rough refined model remain unchanged. For example, models would be evaluated using LID, sGO, and redundancy, then degree centrality, sGO, and redundancy, and so on. The final refined model is generated after this second round of feature selection in which we further reduce the number of *Cv* features used, an additional 25 versions of the candidate model were generated, each containing only one *Cv* feature each while keeping all other features the same. Initial analysis showed that a single *Cv* measure, the local interaction density (LID), could be used in place of all other *Cv* measures without reducing prediction performance of Refined models. This result was not surprising since most *Cv* metrics are highly correlated or anti-correlated with each other in the budding yeast PPI network (Supplementary Figure 3A). Therefore, additional topological metrics will not contribute further improvement to the model’s predictive value beyond LID. Finally, a similar feature selection procedure was applied on three different homology features and results showed that percent amino acid identity had a strong enough individual effect on model performance that the other features were not needed. As with topological measurements, this result is unsurprising given the almost perfect correlation between homology features (Supplementary Figure 3B).

***Generalization***

In generalization experiments, the model is trained on the entire budding yeast dataset and evaluated on the target organism’s (*S. pombe*, *H. sapiens*, or *D. melanogaster*) entire dataset. To reduce variability in model performance, an ensemble of 10 yeast-trained models were used. These models are trained on 10 splits of the yeast single or double gene disruption datasets, each with a different validation set for early stopping. The final predictions of the ensemble are obtained by simply averaging the predictions of the 10 models. It is worth noting that, unlike cross-validation experiments, there is no repeated training/testing splits here. The models get trained on one training set (budding yeast) and tested on one test set (three organisms).

***Interpretation***

To determine the fitted parameter values of the MN models and their confidence intervals, the Bootstrap method was used. Here, the training data is sampled randomly with replacement (meaning that the same training instance can appear multiple times). The model is trained without early stopping for a pre-specified number of epochs (500, 150, and 50 epochs for single gene disruption CA, CA/MO, and GI datasets, respectively) and the fitted parameter values at the end are recorded. This procedure was repeated 50 times resulting in 50 sets of fitted coefficient values for a given dataset. The mean and standard error of the mean was then calculated for each coefficient. Based on these two values, the 95% confidence interval (CI) of each coefficient can be computed using a Student’s T-distribution with 49 degrees of freedom.

**Comparisons with Other Models**

As stated in the main text, the gene features of a select group of published models were used as inputs to the described NN models. Depending on the published model, either a feed-forward NN or the MN model were used as the modeling tool. A detailed description of the process used to extract the features employed by each published model is provided below.

***Single Mutant Fitness* Models’ Comparisons**

For Campos *et al*.^9^, an R script was created based on the author’s own source code to generate amino acid features for all budding yeast genes. The script uses the *protr* R package to calculate the amino acid descriptor (*extractAAC*), the dipeptide composition descriptor (*extractDC*), the tripeptide composition descriptor (*extractTC*), the conjoint triad descriptor (*extractCTriad*), the composition descriptor of CTD descriptors (*extractCTDC*), the transition descriptor of CTD descriptors (*extractCTDT*), the distribution descriptor of CTD descriptors (*extractCTDD*), the normalized Moreau-Broto Autocorrelation descriptor (*extractMoreauBroto*), the Moran autocorrelation descriptor (*extractMoran*), the Geary autocorrelation descriptor (*extractGeary*), sequence-order-coupling numbers (*extractSOCN*), the Quasi-Sequence-Order (QSO) descriptor (*extractQSO*) with lag of 30 steps, the pseudo amino acid composition descriptor (*extractPAAC*) with a lag of 30 steps, and the Amphiphilic pseudo amino acid composition (*extractAPAAC)* with a lag of 30 steps. This generates 9,920 features which are fed into a feed-forward NN with two hidden layers of 100 and 10 units.

Lou *et al.* ^10^ proposes the Local Interaction Density (LID) centrality metric, which is used in this work, and the In-Degree Centrality (IDC) of the complexes in which the protein participates. They use a simple additive weighting scheme to combine the two centralities in order to predict gene essentiality. To replicate their proposed combined LID-IDC centrality, IDC was implemented using the database of yeast protein complex membership ^11,12^, and two centralities, LID and IDC, were fed into the following logistic regression model:

$$logit\left( p_{Lethal} \right)=\psi_{0}+\psi_{1}LID+\psi_{2}IDC$$

The coefficients $\psi_{0},\psi_{1},\psi_{2}$ are treated as trainable parameters of the model and are learned based on the training set.

To replicate Mistry *et al.*^13^, their DiffSLC gene essentiality metric, which combines multiple centralities and experimental data, was implemented. DiffSLC uses gene co-expression thus the protein expression matrix, which evaluates protein expression under 36 conditions, was downloaded from the Github repository associated with Mistry *et al.*, and processed by mapping the Affymetrix probe set ID to the corresponding yeast protein locus tag (expression matrix file name: *Full_RMA_Processed_GSE3431_Expression_Matrix.RDS*). To determine co-expression based on this matrix, DiffSLC implementation was used to calculated the distance correlation metric ^13^ between the expression profiles of all pairs of yeast genes via the *dcor* python package. Given this co-expression matrix, DiffSLC first computes the biased degree centrality (BDC) of all proteins and then combines it with eigenvector centrality via a weighting scheme that involves two weights, $\beta$ and $\omega$. Based on the author’s sweep of the values of those two parameters, the following values were set: $\beta=0.3$ and $\omega=0.9$. This combination of values had the highest AUC-ROC in the unweighted PPI networks (see sheet 1 of Supplementary Table 2 of Mistry *et al.* 2017 ^13^). Finally, we feed the DiffSLC centrality to the following logistic regression model to predict gene essentiality:

$$logit\left( p_{Lethal} \right)=\theta_{0}+\theta_{1}DiffSLC$$

***Negative GI* Models’ Comparisons**

Implementing the SLant model proposed by Benstead-Hume *et al.* ^14^ was straightforward as almost all the input features proposed in that work were used in this work as well. Specifically, all the PPI-based metrics that characterize single genes and pairs of genes were used. Following Slant, the number of shared GO terms between pairs of genes in three aspects (biological process, cellular compartment, and molecular function) were also included. All of those features were fed into the same Siamese NN architecture used for the models described in this work.

For comparison with the model described in Yu *et al*. ^15^, their method, which uses a representation that counts, for each GO term, the number of genes in the pair that have that term (so each term is associated with a count of 0, 1, or 2) was implemented. Following the authors’ methodology, the full set of GO terms (about 6,000 terms) were used when performing this calculation. Therefore, each pair of genes in the GI dataset was associated with an input feature vector of 6,000 elements. These features were fed to a feed-forward NN with two hidden tanh layers of 100 and 10 units, respectively.

The Adjusted-Czekanowski–Dice Dissimilarity (ACDD) metric from Alanis-Lobato *et al*. ^16^ was also implemented. This model was reported to have the highest performance in terms of Area Under the Precision Curve (AUP) on the yeast GI network. The author’s setup is different than the setup described in this work as they attempt to perform link prediction based on the existing GI network. In this work, the existing GI network is not used. Rather an attempt is made to predict GIs based on input features. Nonetheless, since ACDD relies only on network features, it was calculated using the yeast PPI network for all pairs of genes in the GI dataset and fed into a logistic regression model to predict negative GI versus everything else:

$$logit\left( p_{negative} \right)=\nu_{0}+\nu_{1}ACDD$$

**Independent Variables (Input Features)**

***Protein-protein co-precipitation network (PPC)***

Large-scale protein interaction networks have been generated using a variety of methods, including yeast two-hybrid (Y2H) techniques, protein-fragment complementation assays (PCA), and affinity purification followed by mass spectrometry (AP/MS). Each of these approaches detect interactions in a distinct manner, Y2H and PCA can detect direct binary and potentially transient interactions, while the AP/MS method detects stable and abundant protein complexes without necessarily elucidating the specific proteins’ binding patterns. However, Y2H has a high rate of false positives and negatives that, by some estimations, can reach up to 70% of total observations within a dataset ^17,18^, while the AP/MS method is known to detect stable and abundant protein complexes that exist *in vivo* ^19,20^, but cannot necessarily identify the direct contact points between proteins that co-precipitate with one another. Therefore, to avoid a high level of reported interactions based on false positive experimental results, protein-protein networks for the four organisms analyzed were constructed using data derived solely from AP/MS methods and previously published reconstituted protein complexes. Specifically, data obtained from the following techniques was extracted and used from the BioGRID dataset ^21^: Affinity Capture, Affinity Capture Western, Co-fractionation, Co-purification, Reconstituted Complex, Co-crystal Structure, and Protein Peptide, where each dataset contains a list of pairs of genes which were found to co-precipitate under the corresponding experimental technique. These pairwise lists were used to form the edges and nodes of the PPC network in each organism analyzed.

Upon assembling the PPC network in budding yeast, results showed that close to 75% of genes encode proteins that are physically connected to one another forming what is referred to by network scientists as the giant component (GC). The GC is a maximally connected subgraph: a collection of nodes that are directly or indirectly connected to one another but have no connections to other components (typically referred to as islands)^22^. Because of the relatively large size of the GC, the yeast PPC network was limited to the GC only. However, unlike yeast, the GC size in the other organisms is much smaller (40% in *S. pombe* and 20% in *H. sapiens* and *D. melanogaster*), so the PPC networks in those organisms was not constrained to the GC. All in all, the final *S. cerevisiae*, *S. pombe*, *H. sapiens*, and *D. melanogaster* PPC networks contain proteins encoded by 5083, 5129, 46,810, and 18,051 genes, respectively. All of the analyses in this paper are restricted to those sets of genes.

The topological location of each protein in the GC was characterized using the centrality measure *Cv*. The *Cv* of a node is a measure of its importance to network output ^22^. There are many types of *Cv*, each focusing on a certain topological attribute. For example, the *Cv* known as degree centrality is simply the number of direct connections a node has, while the *Cv* known as betweenness centrality is the number of shortest paths between all node pairs within the network that pass through the node of interest. A mixture of software libraries was used along with custom implementations to calculate 23 centralities. Specifically, Python’s networkx 2.3 library was used to calculate degree, eigenvector, subgraph, load, harmonic, Katz, clustering, and information centralities. The igraph 0.8.2 library was used to calculate closeness, betweenness, constraint, coreness, eccentricity, hub score, and neighborhood size centralities. Finally, custom implementations were used to calculate the edge clustering coefficient, local interaction density, maximum neighborhood component, local average connectivity, Lobby index, cutpoint, Harary, and complexity index centralities.

***Protein phosphorylation network***

The most comprehensively examined post-translational modification is protein phosphorylation. This is largely due to the availability of many analytical tools that can detect phosphorylation events and the large number of proteins known to be modified by phosphorylation. The kinase and phosphatase datasets generated from budding yeast provided by Breitkreutz *et al*. ^23^ were used to compute two simple input features: one reflecting phosphorylation events carried out by all known yeast kinases, and the other reflecting dephosphorylation events carried out by all known yeast phosphatases. Specifically, the following two parameters were extracted: the degree-in for a target protein being phosphorylated or dephosphorylated, and the degree-out representing the number of phosphorylation or dephosphorylation events for which a given kinase or phosphatase is responsible.

***Transcriptional network***

Some forms of transcriptional networks are generated by determining the occupancy of transcriptional regulators on the promoters or regulatory elements of a target gene’s sequence. In budding yeast, the genome occupancy of transcription factors, chromatin regulators, general transcriptional machinery, and elongation factors have been determined by Venters *et al*. ^24^. The data from Venters *et al.* (43) was used to compute a simple directed network in which nodes represent genes and edges represent occupancy relationships. For each gene in the network, the following two parameters were extracted: the degree-in, which represents the number of transcriptional regulators that bind to the gene’s sequence, and the degree-out, which represents the number of genes regulated by the transcriptional regulator of interest.

***Protein abundance and localization***

Some computational models incorporate mRNA transcript levels for genes of interest as a proxy for expression levels of the protein encoded by that transcript. However, the amount of transcribed mRNAs is not necessarily linearly correlated with the levels of translated proteins. Thus, a better indicator of protein expression levels for a given gene is direct analysis of the levels of the protein of interest. A collection of yeast strains in which the open reading frames of the majority of genes carry an in-frame COOH-terminal insertion of the Green Fluorescent Protein (GFP) has been generated. Such a collection can be used as a tool to easily assess the expression levels of the protein encoded by the gene fused to GFP. Furthermore, the collection can be used to assess variations in the expression level and/or subcellular localization of a given gene under different growth conditions ^25^. Using these data a matrix was generated where the rows and columns contain lists of the GFP-fused genes and the various cellular compartments in which the protein products of said genes are localized, respectively. The GFP intensity of each gene in each compartment was then registered, and the readings were z-scored by column so that they have a zero mean and a unit standard deviation. A similar matrix was generated for the changes in GFP levels or GFP signal localization at different time points following treatment of the cells with either rapamycin or hydroxyurea. All of these matrices were used as input features for model construction.

***Slim gene ontology***

Gene Ontology (GO) is a characterization of a gene product (mostly proteins, but occasionally RNA) that is derived from experimental observations ^21,26^. Based on these observations, genes are broadly placed into three main domains, the first of which is the biological processes domain, which refers to the cellular process in which the gene product is involved. Such cellular processes are typically carried out by multiple genes and pathways with different molecular activities. In yeast for example, the cell cycle involves the activities of many molecular players involved in different pathways. Such players include DNA polymerase, cyclin-dependent kinases, proteasome, etc. The second domain is the cellular compartment domain, which refers to the cellular compartment in which the gene product is located and in which it carries out its function. For example, DNA polymerase is located in the nucleus. Finally, the third domain is the molecular-level activity domain, which refers to the molecular function of the gene products. For example, the DNA polymerase from the previous two examples is an enzyme that synthesizes DNA molecules from deoxyribonucleotides, the building blocks of DNA.

The full set of GO term annotations can be extensive reaching up to several thousand, containing both fine-grained and species-specific terms. This makes the description of a given gene with the full set of GO terms not only laborious, but too specific to be useful for the construction of a generalized computational model. Thus, slimmed-down versions of GO annotations have been generated providing a broad overview without the species- or gene-specific fine-grained terms. Such slimmed-down versions are referred to as slim GO (sGO) annotations. The budding yeast’s sGO annotations from *yeastgenome.com* ^27^ were used. For the other three organism’s, sGO annotations were generated by feeding their GO annotations into the map2slim tool, both of which are available from geneontology.org ^26^. The sGO features were then limited to the common set of 45 sGO terms found in the *S. cerevisiae*, *S. pombe*, *H. sapiens*, and *D. melanogaster* sGO lists. Using this set, a matrix was created where the rows and columns correspond to genes and sGO terms, respectively. Cells in the matrix indicate whether a gene is associated with a particular sGO term (1) or not (0). In summary, each gene is characterized by 45 binary features which indicate the presence or absence of a given sGO term.

***Gene homology within the genome***

Another input feature used in our model is whether the organism’s genome encoded proteins that can partially or fully replace the function of the protein encoded by the knocked-out gene (i.e. whether there is a spare part or not). This feature was computed using basic local alignment search tool for proteins (BLASTp) for all knocked-out proteins against the entire proteome of each of the four organisms as provided by the National Center for Biotechnology Information (NCBI). Blastp was performed with an e-value cutoff of 0.01 to reduce the possibility of random matches. For each protein encoded by the knocked-out gene, the closest protein in the PPC network in terms of sequence similarity was determined. Then, three statistical metrics were used to encode the similarity of the two proteins: percent identity (how many amino acids exactly match), percent positive (how many amino acids are similar), and the bit score (how good the match is). These statistics are then taken and used as input features characterizing the redundancy of a specific protein).

***Pairwise Features for GI Analysis***

For GI analysis, an additional PPC-based set of features was created that jointly characterize the target gene pair. The set includes (a) shortest path length in the PPC network between the proteins encoded by the target gene pair, (b) node adhesion, defined as the minimum number of edges that must be removed from the PPC network to disconnect the two vertices into two separate components ^14,28^, (c) node cohesion, defined as the minimum number of nodes that must be removed from the PPC network to disconnect the two vertices into two separate components ^14,28^, (d) the number of mutual neighbors between a pair of vertices in the PPC network ^14,28^, and (e) adjacency, which refers to whether two nodes in the PPC network are directly connected by an edge.

**Dependent variables (phenotypes/output)**

***Data Sources***

For each genotype of budding yeast, colony size was used as a proxy for the fitness of single, double, and triple gene disruptions ^29^; BioGRID ^21^ data sets were also used to construct a hybrid dataset that combines manually curated genetic interactions in BioGRID with all the neutral interactions from the Costanzo *et al.* dataset. For triple GIs in budding yeast, the dataset from Kuzmin *et al.* ^30^ was used. For *S. pombe*, the dataset from Malecki and Bahler ^31^ was used along with the list of essential genes in *S. pombe* ^32^. For single mutant fitness and for GIs, a hybrid dataset based on BioGRID and neutral GIs from Ryan *et al*. ^21,33^ was constructed. For *H. sapiens*, the data from Wang *et al*. ^34^ was used for single mutant fitness, while the GI dataset was constructed from interactions reported in BioGRID ^21^ and randomly-sampled neutral interactions. For *D. melanogaster*, the data from Viswanatha *et al.* ^35^ was used for single mutant fitness while the data from FlyBase’s ^36^ list of genetic interactions was used to construct a GI dataset with neutral interactions sampled randomly.

***Binning Scheme for Single-Gene Disruptions***

For single gene disruptions, the continuous phenotype readings were subdivided into three bins: lethal, reduced growth, and normal. The lethal bin contained all genes deemed to be essential for the organism. However, the criteria for the reduced growth and normal bins were different for different organisms. In the budding and fission yeasts, a gene knockout was deemed to yield normal growth if the relative colony size had more than 20% probability of being greater than or equal to 1; otherwise, the gene’s knockout was deemed to yield reduced growth. These probabilities can be computed because these single knockout experiments were repeated and thus a standard deviation is calculable. In *H. sapiens*, genes were classified as lethal if they had a CRISPR score < -2 and p-value < 0.05; genes were classified as reduced growth genes if they had a CRISPR score > -2 and < -1, and p-value < 0.05; and genes were classified as normal growth genes if they had a CRISPR score > -1. Finally, in *D. melanogaster*, normal growth genes were those whose knockout produces an sgRNA level > 0 with a probability of at least 20%, otherwise the genes were classified as having reduced growth phenotypes (if they are not lethal). In binary single mutant fitness analyses, the reduced growth and normal classes were combined to form one class, yielding a lethal versus viable subdivision. Table 1 reports the number of samples and class breakdown of single mutant fitness datasets in all organisms.

Table 1: Single mutant fitness dataset statistics

| Organism | Lethal | Reduced Growth | Normal |
| --- | --- | --- | --- |
| Budding yeast | 839 | 994 | 2,487 |
| Fission yeast | 1,243 | 386 | 2,818 |
| Human | 586 | 951 | 15,284 |
| Fruit fly | 1,214 | 4,007 | 8,306 |

***Multicellular Organismal Single Gene Disruption***

For the cell autonomous and multicellular organismal lethal single-gene disruption dataset in *H. sapiens*, the data from Karczewski *et al*. ^37^ was used to extract the multi-organismal lethal (MO) and viable classes. The viable class consists of genes listed in Supplementary Table 7 in the above stated paper, where at least one homozygous individual carrying a polymorphism that causes an early stop codon does not exhibit any medical issues. The potential MO lethal class consists of genes that scored in the lowest 10% (1,920 genes) according to the Loss-of-Function Observed/Expected Upper Bound Fraction (LOEUF) metric, which measures the intolerance of genes to deactivation (lower values indicate higher intolerance). By subtracting the CA lethal genes identified in human cell lines by *Shalem* *et al*. ^38^ from the potential lethal class reported by Karczewski *et al.* ^37^, a more definitive list of MO lethal human genes was generated.

The CA and MO dataset in *D. melanogaster* was constructed via a similar subtraction procedure. First, the allele phenotypic data for *D. melanogaster*, timestamped March 2020, was downloaded from FlyBase. Then, a list of potential MO lethal genes was formed by selecting rows where the phenotype column starts with the word “lethal” but does not contain the phrase “with GAL4”, to exclude entries where lethality is caused by overexpression of a normal copy or RNAi version of the gene. This analysis generates a list of 2,756 genes, and after excluding the CA genes identified in fly cell lines by Viswanatha *et al*. ^39^, the list of potential MO-lethal genes reduces to 2,281 genes. Finally, the viable class is created by including all the protein-coding genes in the fly PPC network that are neither CA nor MO lethal. Table 2 reports the cellular and multi-organismal single mutant fitness class breakdown for both humans and fruit flies.

Table 2: Cellular and multi-organismal single mutant fitness dataset statistics

| Organism | Cellular Autonomous | Multi Organismal Lethal | Viable |
| --- | --- | --- | --- |
| Human | 586 | 1,524 | 1,675 |
| Fruit fly | 1,214 | 2,281 | 10,148 |

***Budding Yeast Genetic Interaction Datasets***

As stated earlier, in the budding yeast the Costanzo GI dataset was used and a hybrid dataset was created based on data from BioGRID and Costanzo *et al*. ^29,40^. Traditional knockout mutations in essential genes cause lethality. Thus, using these alleles in the generation of double mutants will not produce viable cells in which GIs can be examined. To circumvent this issue, Costanzo et al.^29^ used temperature sensitive mutant alleles of these genes to generate a double mutant cell that, in the absence of GIs, can grow at 26^o^C. The resulting dataset was then subjected to several filters: The first excludes entries that have alleles of essential genes that are not temperature sensitive. The second only includes gene pair mutants grown at 26^o^C. The third filter excludes gene pairs in which one or both genes are not in the PPI network used in this study (which has 5,083 genes out of approximately 6,275). This preprocessing yields a dataset with 18,042,401 observations. To construct the final Costanzo GI dataset, double-gene disruptions at 26^o^C were classified as negative, neutral, positive, or suppression as follows: the negative class included reliable scores less than -0.08 and the positive class included reliable scores greater than 0.08 (at p-value < 0.05), as recommended by Costanzo. Given that the interpretation of the signaling relationship between a pair of genes producing suppression GIs differs from the interpretation of the signaling relationship between a pair of genes that produce positive GIs, it was necessary to separate the suppression class from the positive class. The suppression GI class can be distinguished by the fact that the raw colony size of the double mutant is greater than the colony size of either single mutant alone. GI scores that do not meet the criteria of any of the negative, positive, and suppression classes were classified as neutral. Furthermore, it was essential to ensure that there were no overlaps between any of the four GI classes.

In the BioGRID dataset, interaction types were mapped as follows: The negative GI class contains Synthetic Lethality, Synthetic Growth Defect, Negative GI, Dosage Growth Defect, Dosage Lethality, and Phenotypic Enhancement interaction types. The positive GI class contains the Positive GI type, and the suppression class contains the Phenotypic Suppression, Dosage Rescue, and Synthetic Rescue interaction types. For the budding yeast, the dataset was filtered by only considering interactions reported in at least 3 publications. Also, since a gene pair can appear in multiple GI bins, the pair was included if it was reported more often in one of the GI bins over others. Since BioGRID does not contain neutral interactions, the GIs from BioGRID were combined with all the neutral class from the Costanzo dataset, regardless of temperature (26^o^C or 30^o^C) such that a pair is considered to be neutral if it is not found in BioGRID. Table 3 reports the class breakdown for both GI datasets in the budding yeast.

Table 3: Genetic interaction dataset statistics

| Organism | Negative | Neutral | Positive | Suppression |
| --- | --- | --- | --- | --- |
| Budding yeast (Costanzo) | 83,988 | 1,936,024 | 42,018 | 6,292 |
| Budding yeast (Hybrid) | 19,638 | 6,897,562 | 1,024 | 646 |
| Fission yeast | 4,271 | 996,862 | 1,474 | 276 |
| Human | 820 | 1,000,000 | 9 | 87 |
| Fruit fly | 3,227 | 1,000,000 | N/A | 5,712 |

Overall, 81%, 71%, and 0% of the negative, positive, and suppression pairs, respectively in the BioGRID dataset were also reported in the Costanzo dataset.

***Budding Yeast Triple Genetic Interaction Dataset***

The triple GI dataset in budding yeast generated by Kuzmin *et al*. ^30^ was used. The dataset originally contained close to 200,000 triplets, but the authors filtered the positive and suppression interactions, resulting in 91,000 triplets. Triplets were deemed to be negatively interacting if they have an adjusted interaction score of less than -0.08 (with a p-value of < 0.05). Based on these values, 2835 observations are negative and 71,628 are neutral.

***Genetic Interactions in Other Organisms***

To generate the GI dataset in *S. pombe*, a similar approach to the hybrid GI dataset construction in budding yeast was used. Specifically, the interactions in the three classes (negative, positive, and suppression) in the BioGRID dataset (using the above-mentioned interaction type mapping with a minimum publication threshold of 2) was used. Then, these interactions were combined with the neutral interactions reported in ^21,33^, which were defined as those with an epistatic miniarray profile (E-MAP) value within the middle 80% of the values. Finally, any pairs with missing single mutant fitness readings in either gene in the pair were eliminated.

For the GI dataset in *H. sapiens*, all interacting pairs were also extracted using the above-mentioned criteria from BioGRID, but the publication threshold criterion was removed to obtain a larger number of observations. To form the neutral class, a set of 1,000,000 gene pairs from the human PPC network were randomly sampled and subjected to the constraints that the pair does not appear in the BioGRID dataset and that both genes in the pair have a single mutant fitness phenotype from the human CRISPR dataset. Due to the low number of positive and suppression GIs (see Table 3), the negative, positive, and suppression classes were combined into one class with the label “interacting”.

The GIs in *D. melanogaster* were extracted from the FlyBase ^36^ dataset (gene genetic interactions dataset, timestamped January 2020). The dataset only contains enhancing and suppression interactions. As with BioGRID, FlyBase does not report neutral interactions, so 1,000,000 gene pairs from the fly PPC network were randomly sampled and subjected to the conditions that they do not appear in FlyBase and that all genes in all pairs have single mutant fitness readings from the fly CRISPR dataset. As with the *H. sapiens* dataset, the enhancing and suppression classes were pooled together to form one class representing GIs. Table 3 reports the class breakdown for the GI dataset in fruit flies.

As stated earlier, the single- and triple-mutant datasets were both split in a class-stratified fashion, thereby preserving the ratios of classes in both the training and testing sets. However, the double-mutant dataset was split in a gene-stratified fashion to ensure that testing examples do not include genes that were seen during training (this wasn’t possible with the triple dataset as the number of samples is too small). Therefore, it would be prudent to check that the class balance is similar between the training and testing portions of the double gene GI datasets. Table 4 shows that this indeed is the case.

Table 4: Average number of samples and their class distribution in the training and testing portions of the GI datasets

|  | Training % | | | | Testing % | | | |
| --- | --- | --- | --- | --- | --- | --- | --- | --- |
|  | - | N | + | S | - | N | + | S |
| Budding yeast (Costanzo) | 4.06 | 93.61 | 2.03 | 0.3 | 4.05 | 93.61 | 2.03 | 0.3 |
| Budding yeast (Hybrid) | 0.29 | 99.69 | 0.01 | 0.01 | 0.28 | 99.69 | 0.01 | 0.01 |
| Fission yeast | 0.43 | 99.40 | 0.15 | 0.03 | 0.43 | 99.40 | 0.15 | 0.03 |
| Human | 0.08 | 99.91 | 0.00 | 0.01 | 0.08 | 99.91 | 0.00 | 0.01 |
| Fruit fly | 0.32 | 99.11 | 0 | 0.57 | 0.32 | 99.11 | 0 | 0.57 |

**Statistical Analyses**

***Models’ Cross Validation Performance***

When performing cross-validation on a model, the model is trained and tested N=50 and N=40 times in single and double/triple gene disruption datasets, respectively. From each run, the overall balanced accuracy on the test set is calculated. To evaluate whether there is a difference in the mean overall balanced accuracy between two models, a paired t-test was used. In the case of single gene disruption, the test compares two lists of N=50 numbers each, while in double/triple gene disruption, the test compares two lists of N=40 numbers each. To account for multiple comparisons among models, the Bonferroni adjustment on the p-value thresholds was applied. Standard p-value thresholds are 0.05 (*), 0.01 (**), 0.001 (***), and 0.0001 (****). With the Bonferroni adjustment, these thresholds are divided by the number of pairwise comparisons being made, denoted as C.

***Violin Feature Plots***

For yeast, the distributions of LID and percent identity scores in the single gene disruption output bins (lethal, reduced growth, and normal) are plotted in Figures 2C and 2D. In Figure 3D, the sum LID scores in the double gene disruption output bins (negative, neutral, positive, and suppression) are plotted. To assess differences between the distributions of a feature in two output bins, the Kruskal-Wallis test is used. This tests whether or not the medians of the two distributions are equal. To account for multiple comparisons among distributions in the output bins, the Bonferroni correction was applied to the standard p-value thresholds: 0.05 (*), 0.01 (**), 0.001 (***), and 0.0001 (****). The Bonferroni correction divides these thresholds by the number of comparisons being made, which is denoted as C. In Figures 2C and 2D, there are C=3 pairwise comparisons among the Lethal (N=839), Reduced Growth (N=994), and Normal (N=2,487) classes. In Figure 3D, there are C=6 pairwise comparisons among the Negative (N=19,638), Neutral (N=6,897,562), Positive (N=1,024), and Suppression (N=646) classes.

***Correlation Among sGO Terms***

In Figure 2B, the frequency of each sGO term in each single gene disruption output bin (lethal, reduced growth, and normal) was computed, generating 3 numbers per sGO term, which sum to 100%. To establish whether there is a pattern in the prevalence of sGO terms in the different output bins, the Spearman-r correlation between the sGO term frequencies in each pair of output bins was evaluated, yielding a total of 3 comparisons between pairs of lists which contain N=45 numbers each (corresponding to 45 sGO terms).

***Generation of the sGO Interaction Matrices***

To generate the sGO interaction matrices in Figures 3E-G, the yeast hybrid GI dataset was processed as follows: First, an empty matrix is initialized for each pair of sGO terms. This matrix will contain values representing how often each pair occurs in each output bin. For every observation in the dataset, the sGO terms associated with both genes in the observation are identified. Then, a set of all possible combinations of those terms is enumerated and for each combination the count in the matrix is incrementally increased by one. Finally, the counts in the matrices are normalized across the negative, positive, and suppression bins as follows:

$$P_{neg}\left( sGO_{a},sGO_{b} \right)=\frac{Count_{neg}\left( sGO_{a},sGO_{b} \right)}{\sum_{bin=\{neg,pos,supp\}208} Count_{bin}(sGO_{a},sGO_{b})}$$

$$P_{pos}\left( sGO_{a},sGO_{b} \right)=\frac{Count_{pos}\left( sGO_{a},sGO_{b} \right)}{\sum_{bin=\{neg,pos,supp\}} Count_{bin}(sGO_{a},sGO_{b})}$$

$$P_{supp}\left( sGO_{a},sGO_{b} \right)=\frac{Count_{supp}\left( sGO_{a},sGO_{b} \right)}{\sum_{bin=\{neg,pos,supp\}} Count_{bin}(sGO_{a},sGO_{b})}$$

***sGO Interaction Matrices Visualization***

To improve the visualization of sGO matrices, the color intensity is manipulated in a non-linear fashion. First, for each cell in the matrix, the values are normalized such that they lie between 0 (minimum) and 1 (maximum). Then, the normalized values are transformed through the following power-law function:

$$y\left( x \right)=1.125 \times(1-\exp\left( -2.1972x \right))$$

The values of the coefficients in the function were chosen such that $y\left( 0.5 \right)=0.75$. This function suppresses differences between values of x close to 1.0 and amplifies differences between values of x close to 0.0. This manipulation is only done for visualization purposes, and not for statistical testing.

***Diagonal versus Off Diagonal in the sGO Matrices***

Diagonal versus off-diagonal entries in Figures 3E-G were compared as follows: First, a vector of the diagonal entries in the matrix is generated. Second, for each column in the matrix, the average off-diagonal value in that column is computed. This process generates two lists: the diagonal values and the average off-diagonal values (both have N=45 elements). Then, a paired t-test is used to determine whether or not the two lists have identical average values.

***Distributions of Shortest Path Lengths***

In Figure 3C, the distribution of shortest path lengths in the yeast PPC network was computed for each output bin in the hybrid GI dataset. In order to assess whether the medians of the distributions of shortest path lengths in two output bins are significantly different, a Kruskal-Wallis test was used. Here, the negative bin has N=19,638 observations, the neutral bin has N=6,897,562 observations, the positive bin has N=1,024 observations, and the suppression bin has N=646 observations. To account for multiple comparisons between all pairs of output bins, the Bonferroni correction was applied on the standard p-values thresholds: 0.05 (*), 0.01 (**), 0.001 (***), and 0.0001 (****). The Bonferroni correction divides these thresholds by the number of pairwise comparisons being made, which is C = 6.

***Molecular Complex and Pathway Analysis***

Molecular complexes refer to protein subunits that physically interact with one another to carry out a given molecular function; an example of such a complex is the proteasome ^32^. A pathway, on the other hand, consists of more than one molecular complex, with the signaling product of one complex conveying its output to another; an example is the mitogen-activated protein kinase (MAPK) pathway ^41^. Curated protein complex data for budding yeast was obtained from the Munich Information Center of Protein Sequences (MIPS) ^11,12^ while data pertaining to pathways was obtained from the Kyoto Encyclopedia of Genes and Genomes (KEGG) ^42^. Four filters were then applied to ensure that an appropriate level of coverage is achieved: The first filter selected complexes and pathways for which at least 25% of genes had been examined experimentally for possible GIs, thus ensuring that any conclusions reached were broadly representative of the general behavior of the components of a given complex or pathway rather than being based only on a few genes. These selected complexes and pathways were then passed into a second filter that selected only ones in which the GI patterns were examined with at least 25% of all the genes present in the budding yeast genome, thus accounting for the background level of genetic interactions of target genes. The third filter eliminated the few genes assigned to more than one pathway or complex to further simplify the analysis. The final filter was placed to distinguish between gene pairs that belong to the same pathway, but do not encode components of the same complex, thus avoiding counting the same GIs multiple times (for a summarized list of selected molecular complexes and pathways and the different types of interactions between different complex units, see Supplementary Table 5 sheets 1 and 2, and Supplementary Figure 6B-C).

Applying these four filters yielded 198 molecular complexes (with an average of about 2,000 gene pairs per complex) and 36 pathways (with an average of about 5,500 gene pairs per pathway; Supplementary Table 5 sheets 1 and 2). The types of GIs most frequently observed within or across molecular complexes or pathways were then examined.

For each molecular complex/pathway in Supplementary Figure 6, there is an associated distribution of within- and across-complex/pathway observations over the four GI categories (Negative, Neutral, Positive, and Suppression). For each category, the presence of a statistical difference in the frequency of within- and across-complex/pathway observations in that category were assessed using a paired t-test with N=198 for complexes and N=36 for pathways.

Less than 5% of gene pairs that encode proteins which are not part of the same molecular complex or pathway show GIs with each other. This proportion significantly increases if the gene pair encodes proteins that are part of the same complex or pathway (40% and 20%, respectively; Supplementary Figure 6B and 6C, yellow). The majority of these within-complex and within-pathway GIs are of the negative type (close to 22% of within complex pairs, and 15% within pathway pairs; Supplementary Figure 6B and 6C, red). Positive GIs accounted for close to 8% and are largely restricted to within complexes (Supplementary Figure 6B and C, green). Finally, suppression GIs represent close to 8% of within-complex examinations and 4% of within pathway examinations (Supplementary Figure 6B and 6C, blue). All of the above indicates that gene pairs which encode components of a single complex, and to a lesser extent a single pathway, were more likely to interact than gene pairs which did not, with the majority of GIs being of the negative type. However, contrary to the traditional interpretation of negative GIs, which stipulates that they occur between gene pairs involved in the same process via separate, redundant complexes or pathways, our results suggest that these GIs often occur due to redundancy within a single complex or pathway. Regarding positive GIs, within-complex gene pairs have a higher probability of being positively interacting than across-complex pairs, supporting the traditional interpretation that positive GIs occur when two genes encode two subunits of the same complex that are both necessary for signaling output. Suppression GIs are predominately observed within genes encoding subunits of the same complex, suggesting auto-inhibition within the complex, and proteins in the same pathway but not the same complex, suggesting inhibition of one complex in the pathway by another complex or its product.

***Feature Distributions of the Triplet GI Dataset***

In Figures 4B, 4C, 4E, 4F, and 4G, the distributions of various features in the negative and neutral classes were plotted. To determine whether those distributions are statistically different, a Chi-squared test was used to calculate statistical significance at p-value thresholds 0.05 (*), 0.01 (**), 0.001 (***), and 0.0001 (****). Statistical significance in the remaining feature plots, 4D and 4E, is assessed as described above.

**References**

1. Stuart J. Russell, P.N. *Artificial Intelligence: A Modern Approach*, (Prentice Hall, USA, 2020).

2. Ian Goodfellow, Y.B., and Aaron Courville. *Deep Learning*, (The MIT Press., Boston, MA, 2016).

3. Goodfellow, I.B., Yoshua; Courville, Aaron *6.2.2.3 Softmax Units for Multinoulli Output Distributions, Deep Learning*, 180–184. (MIT Press, 2016).

4. Jane Bromley, I.G., Yann LeCun, Eduard Sickinger and Roopak Shah. Signature Verification using a "Siamese" Time Delay Neural Network *International Journal of Pattern Recognition and Artificial Intelligence* **7**, 25 (1993).

5. Dozat, T. ncorporating Nesterov Momentum into Adam. in *International Conference on Learning Representations Workshop* (San Juan, Puerto Rico, 2016).

6. Chicco, D. Ten quick tips for machine learning in computational biology. *BioData Min.* **10**(2017).

7. Brodersen, K.H., Ong, C. S., Stephan, K. E., & Buhmann, J. M. . The balanced accuracy and its posterior distribution. . in *The 2010 20th International Conference on Pattern Recognition*  3121-3124 (IEEE, 2010).

8. Velez, D.R., White, B. C., Motsinger, A. A., Bush, W. S., Ritchie, M. D., Williams, S. M., & Moore, J. H. A balanced accuracy function for epistasis modeling in imbalanced datasets using multifactor dimensionality reduction. *Genetic Epidemiology: the Official Publication of the International Genetic Epidemiology Society,* **31**, 306-315. (2007).

9. Campos, T.L., Korhonen, P.K., Sternberg, P.W., Gasser, R.B. & Young, N.D. Predicting gene essentiality in Caenorhabditis elegans by feature engineering and machine-learning. *Comput Struct Biotechnol J* **18**, 1093-1102 (2020).

10. Luo, J. & Wu, J. A new algorithm for essential proteins identification based on the integration of protein complex co-expression information and edge clustering coefficient. *Int J Data Min Bioinform* **12**, 257-74 (2015).

11. Pu, S., Vlasblom, J., Emili, A., Greenblatt, J. & Wodak, S.J. Identifying functional modules in the physical interactome of Saccharomyces cerevisiae. *Proteomics* **7**, 944-60 (2007).

12. Pu, S., Wong, J., Turner, B., Cho, E. & Wodak, S.J. Up-to-date catalogues of yeast protein complexes. *Nucleic Acids Res* **37**, 825-31 (2009).

13. Mistry, D., Wise, R.P. & Dickerson, J.A. DiffSLC: A graph centrality method to detect essential proteins of a protein-protein interaction network. *PLoS One* **12**, e0187091 (2017).

14. Benstead-Hume, G. *et al.* Predicting synthetic lethal interactions using conserved patterns in protein interaction networks. *PLoS Comput Biol* **15**, e1006888 (2019).

15. Yu, M.K. *et al.* Translation of Genotype to Phenotype by a Hierarchy of Cell Subsystems. *Cell Syst* **2**, 77-88 (2016).

16. Alanis-Lobato, G., Cannistraci, C.V. & Ravasi, T. Exploitation of genetic interaction network topology for the prediction of epistatic behavior. *Genomics* **102**, 202-8 (2013).

17. Auerbach D, S.I. Yeast Two-Hybrid Protein-Protein Interaction Networks. *Protein Reviews* **3**, 19–31 (2005).

18. Deane CM, S.Ł., Xenarios I, Eisenberg D (Protein interactions: two methods for assessment of the reliability of high throughput observations. *Molecular & Cellular Proteomics* **1** 349–56 (2002).

19. Gavin, A.C. *et al.* Functional organization of the yeast proteome by systematic analysis of protein complexes. *Nature* **415**, 141-7 (2002).

20. Ho, Y. *et al.* Systematic identification of protein complexes in Saccharomyces cerevisiae by mass spectrometry. *Nature* **415**, 180-3 (2002).

21. Oughtred, R. *et al.* The BioGRID interaction database: 2019 update. *Nucleic Acids Res* **47**, D529-D541 (2019).

22. Newman, M.E.J. *Networks: An Introduction*, ( Oxford University Press, Oxford, UK, 2010).

23. Breitkreutz, A. *et al.* A global protein kinase and phosphatase interaction network in yeast. *Science* **328**, 1043-6 (2010).

24. Venters, B.J. *et al.* A comprehensive genomic binding map of gene and chromatin regulatory proteins in Saccharomyces. *Mol Cell* **41**, 480-92 (2011).

25. Chong, Y.T. *et al.* Yeast Proteome Dynamics from Single Cell Imaging and Automated Analysis. *Cell* **161**, 1413-24 (2015).

26. The Gene Ontology, C. The Gene Ontology Resource: 20 years and still GOing strong. *Nucleic Acids Res* **47**, D330-D338 (2019).

27. Cherry, J.M. *et al.* Saccharomyces Genome Database: the genomics resource of budding yeast. *Nucleic Acids Res* **40**, D700-5 (2012).

28. Nepusz, T. Python-igraph manual (2012).

29. Costanzo, M. *et al.* A global genetic interaction network maps a wiring diagram of cellular function. *Science* **353**(2016).

30. Kuzmin, E. *et al.* Systematic analysis of complex genetic interactions. *Science* **360**(2018).

31. Malecki, M. & Bahler, J. Identifying genes required for respiratory growth of fission yeast. *Wellcome Open Res* **1**, 12 (2016).

32. Tanaka, K. The proteasome: overview of structure and functions. *Proc Jpn Acad Ser B Phys Biol Sci* **85**, 12-36 (2009).

33. Ryan, C.J. *et al.* Hierarchical modularity and the evolution of genetic interactomes across species. *Mol Cell* **46**, 691-704 (2012).

34. Wang, T. *et al.* Identification and characterization of essential genes in the human genome. *Science* **350**, 1096-101 (2015).

35. Viswanatha, R. *et al.* Pooled CRISPR Screens in Drosophila Cells. *Curr Protoc Mol Biol* **129**, e111 (2019).

36. Thurmond, J. *et al.* FlyBase 2.0: the next generation. *Nucleic Acids Res* **47**, D759-D765 (2019).

37. Karczewski, K.J. *et al.* The mutational constraint spectrum quantified from variation in 141,456 humans. *Nature* **581**, 434-443 (2020).

38. Shalem, O. *et al.* Genome-scale CRISPR-Cas9 knockout screening in human cells. *Science* **343**, 84-87 (2014).

39. Viswanatha, R., Li, Z., Hu, Y. & Perrimon, N. Pooled genome-wide CRISPR screening for basal and context-specific fitness gene essentiality in Drosophila cells. *Elife* **7**(2018).

40. Costanzo, M. *et al.* The genetic landscape of a cell. *Science* **327**, 425-31 (2010).

41. Guo, Y.J. *et al.* ERK/MAPK signalling pathway and tumorigenesis. *Exp Ther Med* **19**, 1997-2007 (2020).

42. Du, J. *et al.* KEGG-PATH: Kyoto encyclopedia of genes and genomes-based pathway analysis using a path analysis model. *Mol Biosyst* **10**, 2441-7 (2014).

**Supplementary figures**

**Supplementary Figure 1. The spectrum of the different GI results obtained in budding yeast studies and their conventional interpretation**. In this example, the hypothetical diameter of the budding yeast colony in single or double gene knockout is shown red font. In the left side the impact of a single gene knockout (*A^Δ^* or *B^Δ^* only) is shown. In the right side, all potential scenarios of the impact of double gene knockout (*A^Δ^* and *B^Δ^*) are shown. Genes in the same color box are involved in the same biological process but necessarily the same pathway, with red arrows indicating positive signals and blue lines with flat ends indicating inhibitory ones between genes involved in the same pathway.

**Supplementary Figure 2. Summary of the methodology used to generate the different models developed in this study using single gene fitness models as an example.** A feed-forward NN used to construct the initial black-box model, S-Full (upper panel), in which all input features (top row) are fed into the input neurons *x*_x_ that are connected to the hidden layer neurons *u*_x_ , those hidden neurons are then wired to the output layer composed of three units corresponding to lethal (L), reduced growth (R), and normal growth (N). The input features are as follows: the coprecipitation network consists of proteins (grey nodes) with edges (grey edges) representing physical interactions among them. The phosphorylation network consists of phosphatases (blue circles), kinases (yellow circles), and targets (unfiled blue or yellow circles) with yellow and blue arrows indicating phosphorylation and dephosphorylation relationships, respectively. The transcription network consists of transcription factors (fill red circles) and targets (unfilled red circles) with red arrows representing transcription relationships. Localization features measure the abundance of the protein in various cellular compartments (green) as measured by the GFP signal. The sGO features are binary-coded vectors that indicate the presence (black cell) or absent of a given sGO terms associated with a gene. The homology features are found by executing a BlastP search over the organism’s genome to determine the closest match to a given gene. Red asterisks and blue arrowhead in the training and validation sets correspond to genetic disruptions which cause lethality and reduced growth, respectively. Following the evaluation of the full model, feature selection is used to create the S-Refined model which has the smallest set of input features capable of achieving comparable performance to the S-Full model (middle row). Finally, additional constraints (removal of the hidden layers) result in the S-MN model which enables direct quantitative evaluation of the effect of each input feature on the distribution of output classes. In all three stages, the models are evaluated based on results of repeated cross-validation on a development set for the budding yeast. The final definitive evaluation of the models occurs on a separate test set that is never seen during model development and training.

**Supplementary Figure 3. The custom NN architectures designed to predict the impact of single, double, and triple genetic knockouts on cell viability. (A)** The architecture of a NN that predicts single mutant fitness. Colored rectangles represent distinct sub-NNs that process distinct feature sets. The black arrows on the left indicate the feature sets and the number of features contained within each set. The grey shading corresponds to the weighted connections between layers in the NN. All modules in the model use a standard feed-forward architecture, except the localization module, which applies a feed-forward network to each compartment’s time series (framed in the bottom right). **(B)** The architecture of the double GI-predicting NN. Rectangles with thick borders and the same color correspond to modules which internally share the same weights (e.g., the green rectangles are the same mathematical function applied to two different input feature sets). **(C)** The architecture of the triple GI-predicting neural network. Same as rectangles in **(B),** rectangles in **(C)** with the same color correspond to modules which internally share the same weights (e.g., the green rectangles are the same mathematical function applied to two different input feature sets).

**Supplementary Figure 4. Spearman-*r* correlation scores of the different PPI centrality metrics (A) and BLAST sequence similarity scores (B).**

**Supplementary Figure 5. Performance of computational models predicting four-way GIs in budding and fission yeast. (A)** Performance of the models as measured by average balanced accuracy, confusion matrices, and per-class ROC (with the corresponding AUC-ROC values) over 10 replications of 4-fold cross-validation on the full Costanzo dataset. **(B)** Performance of the models on fission yeast over 10 replications of 4-fold cross-validation. The purple, red, blue, and white colors correspond to the D-Full, D-Refined, D-MN, and null models, respectively. In all panels, the output classes are negative (-), neutral (N), positive (+), and suppression (S). Statistical analysis (error bars and asterisks) is similar to Figure 1 with the asterisk color reflecting the model being compared, with C=6 for panel A and C=3 for panel B.

**Supplementary Figure 6. Magnification of Figure 3D-F along with full sGO term labels.**

**Supplementary Figure 7. Performance of three-way S-Refined and S-MN models when predicting single gene knockout cell autonomous (CA) phenotypes in fission yeast (*S. pombe*), human cell lines (*H. sapiens*), and fruit fly cell lines (*D. melanogaster*).** Cross validation performance of the S-Refined (red), S-MN (blue), and baseline models (white) in *S. pombe* (A), *H. sapiens* (B), and *D. melanogaster* (C). Each panel shows the average balanced accuracy (left), confusion matrices (middle: L, R, and N correspond to lethal, reduced growth, and normal phenotypes, respectively), and per-class ROC (right) over 10 replications of 5-fold cross-validation. Statistical analysis (error bars and asterisks) is similar to figure 2A with the asterisk color reflecting the model being compared and C=3.

**Supplementary Figure 8. Performance of binary S-Refined and S-MN models when predicting CA, MO, and V single-gene knockouts in humans and fruit flies.** Each panel shows the average balanced accuracy, confusion matrices (L for lethal and V for viable), and per-class ROC (with the corresponding AUC-ROC values) over 10 replications of 5-fold cross-validation. Statistical analysis (error bars and asterisks) is similar to figure 2A with the asterisk color reflecting the model being compared and C=3.

**Supplementary Figure 9. Comparison between the S-MN and D-MN models to previously published models on the budding yeast datasets**. Cross-validation performance of all models as measured by average overall balanced accuracy, confusion matrices, and per-class ROC (with the corresponding AUC-ROC values) over repeated cross-validation on the full yeast datasets. **(A)** Three essential gene-predicting models (with their names shown on the balanced accuracy histogram) compared to the S-MN model (blue). **(B)** Three different negative GI-predicting models (with their names shown on the balanced accuracy histogram) compared to the D-MN model (blue) on the yeast hybrid GI dataset. Statistical analysis (error bars and asterisks) is similar to Figure 2A with the asterisk color reflecting the model being compared and C=6.

**Supplementary Figure 10. Cross-prediction experimental setup and performance of the D-MN model.** A) The D-MN model is trained to predict GIs (hybrid dataset), but evaluated (red arrow) on GI (red asterisks and blue arrowhead correspond to genetic disruptions which cause lethality and reduced growth), coprecipitation (blue), phosphorylation (dash yellow arrow), and transcription prediction tasks (dash red arrow). The input features are as follows: the coprecipitation network consists of proteins (grey nodes) with edges (grey edges) representing physical interactions among them. The single mutant fitness represented as a diameter of the budding yeast colony (yellow circle) in single gene knockout (*A^Δ^* or *B^Δ^*, lower panel). Finally, the sGO features as a binary-coded vectors that indicate the presence (black cell) or absent of a given sGO terms associated with a gene. The training and evaluation are done via 10 replications of 4-fold cross validation on the development dataset, as usual. (B) The same experiment is conducted but with a D-MN model (D-MN-Modified) that is missing topology features (greyed out) and three sGO terms: *kinase*, *phosphatase*, and *transcription* binding (red font). (C) Cross-validation performance of the D-MN (solid bars) and D-MN-Modified (hatched bars) models on each of the four prediction tasks. Error bars correspond to standard deviations. Asterisks represent *p*-values, p < $0.05$ (*), p < $0.01$ (**), p <$0.001$ (***), and p <$0.0001$ (****) and indicate the reliability of paired t-tests between the performance of D-MN and D-MN-Modified models.

**Supplementary Figure 11. The contribution of the input features to the MN models is task-specific.** The percentage drop in overall balanced accuracy, as reflected by the height of the bar, of models missing each input feature relative to the regular MN model. The greater the bar height, the more important the corresponding input feature. Error bars correspond to standard deviations over 10 replications of cross-validation. Asterisks represent Bonferroni-corrected *p*-values, p < $\frac{0.05}{C}$ (*), p < $\frac{0.01}{C}$ (**), p <$\frac{0.001}{C}$ (***), and p <$\frac{0.0001}{C}$ (****), where C=3 for single mutant fitness and C=6 for double and triple mutant fitness tasks. Asterisk colors correspond to the models being compared.

**Supplementary Figure 12. Universality of the MN models.** **(A)** The MN models are trained only on the budding yeast datasets then directly tested another organism’s input/output data. **(B-D)** Generalization performance of S-MN model trained only on budding yeast data to predict lethal (L) versus viable (V) single gene knockout phenotypes, as evaluated on the other organisms. **(E-G)** Generalization performance of the D-MN model trained only on the budding yeast hybrid GI dataset to predict interacting (I) versus neutral (N) double gene knockout phenotypes, as evaluated on the three other organisms. The lack of error bars in the figures is due to the models being tested on the organism’s entire dataset in one shot.

**Supplementary Figure 13. A screenshot of web-based service for reporting GIs at different thresholds in all tested organisms. (A)** The search page for discovering double and triple GIs of a target gene or a specific gene pair (upper panel). When the user clicks “Details” the website shows the individual and combined gene features and the components of the D-MN or T-MN model that generate the prediction**. (B)** The search page for discovering common genetic interactors with up to four genes.

**Supplementary tables legends**

**Supplementary Table 1.** Hyperparameter optimization results for the S-Full (Sheet 1) and D-Full (Sheet 2) models on the budding yeast datasets.

**Supplementary Table 2.** Comprehensive feature selection results of models used to predict the impact of single gene knockout on budding yeast colony size.

**Supplementary Table 3.** Mean S-MN model coefficient values and their 95% confidence intervals in all tested organisms for three-way (sheet 1) and binary (sheet 2) classification. The table shows the coefficient values (and exponentiated values). In each of these three classes, a unit increase in input features associated with exponentiated coefficient values greater than 1.0 increases odds of the class over the neutral class, and vice-versa.

**Supplementary Table 4.** Comprehensive feature selection results of models used to predict GIs in budding yeast on the hybrid GI dataset.

**Supplementary Table 5.** List of budding yeast molecular complexes (sheet 1) and pathways (sheet 2) used in generating supplementary figures 2I and J.

**Supplementary Table 6.** Mean D-MN model coefficient values and their 95% confidence intervals in all tested organisms in 4-way (sheet 1) and binary (sheet 2) classification tasks. The table in sheet 1 shows the coefficient values (and exponentiated values) of the negative, positive, and suppression classes, with respect to the neutral class. In each of these three classes, a unit increase in input features associated with exponentiated coefficient values greater than 1.0 increases odds of the class over the neutral class, and vice-versa. The table in sheet 2 shows the coefficient values of the interacting class with respect to the neutral class.

**Supplementary Table 7.** Comprehensive feature selection results of models used to predict budding yeast triple gene knockout GIs.

**Supplementary Table 8.** Mean of budding yeast T-MN model coefficient values and their 95% confidence intervals.
