# Supplementary figures and images for: Predicting and explaining the impact of genetic disruptions and interactions on organismal viability
Source: Bioinformatics. 2022 Jul 21;38(17):4088–99. doi: 10.1093/bioinformatics/btac519 (PMC9438956; doi:10.1093/bioinformatics/btac519)

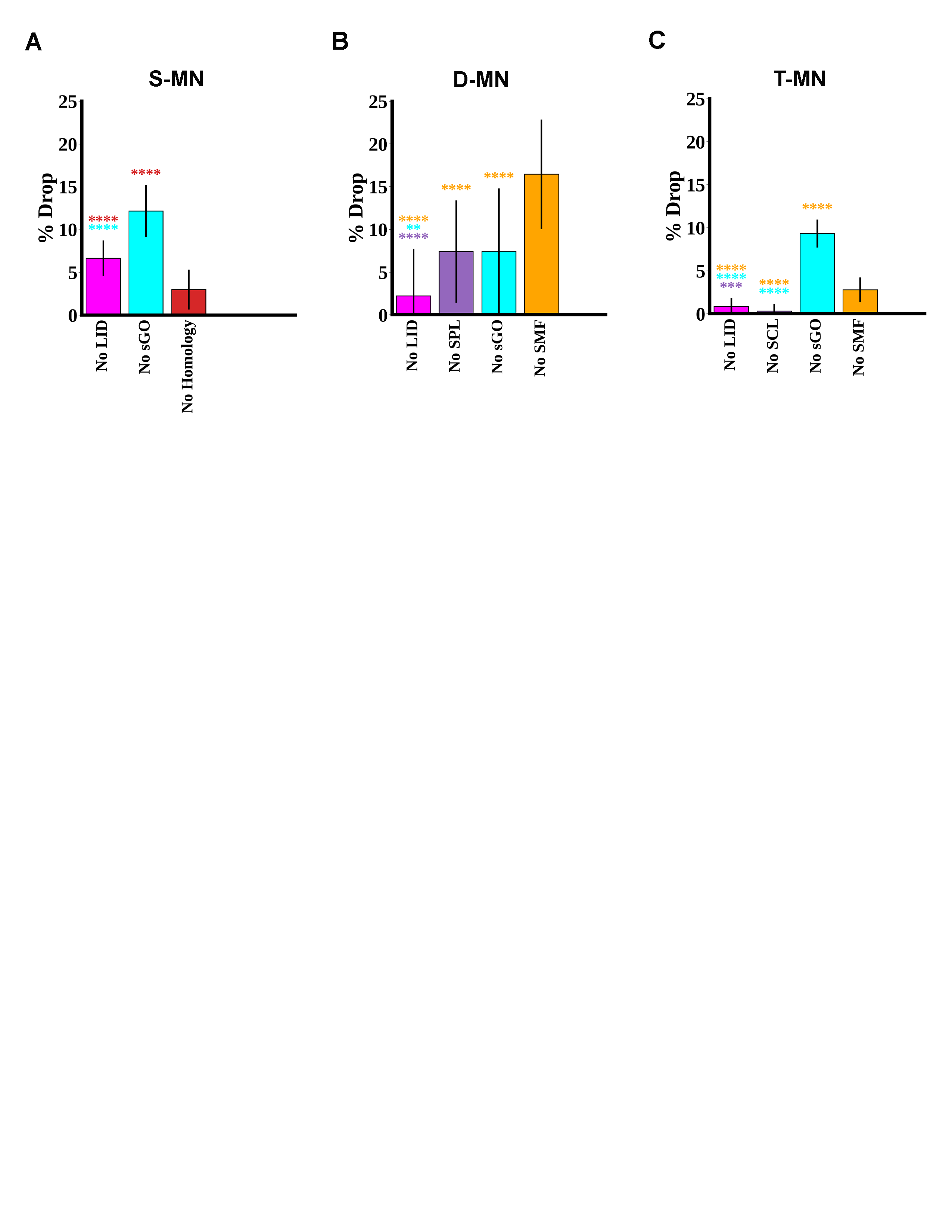

Supplement: btac519_Supplementary_Data [file btac519_supplementary_data.zip › btac519_Supplementary_Data/Supplementary Figure 11.tif]

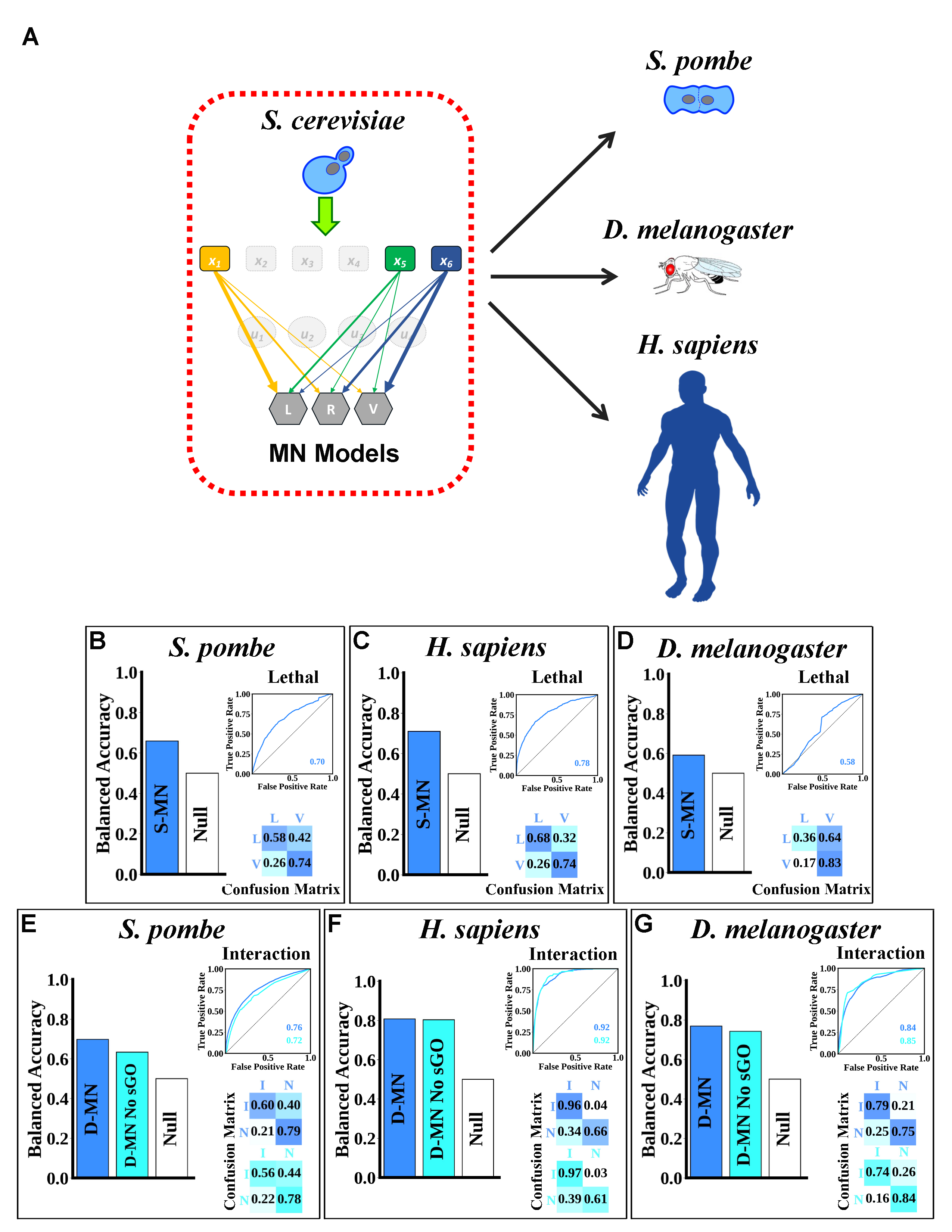

Supplement: btac519_Supplementary_Data [file btac519_supplementary_data.zip › btac519_Supplementary_Data/Supplementary Figure 12.tif]

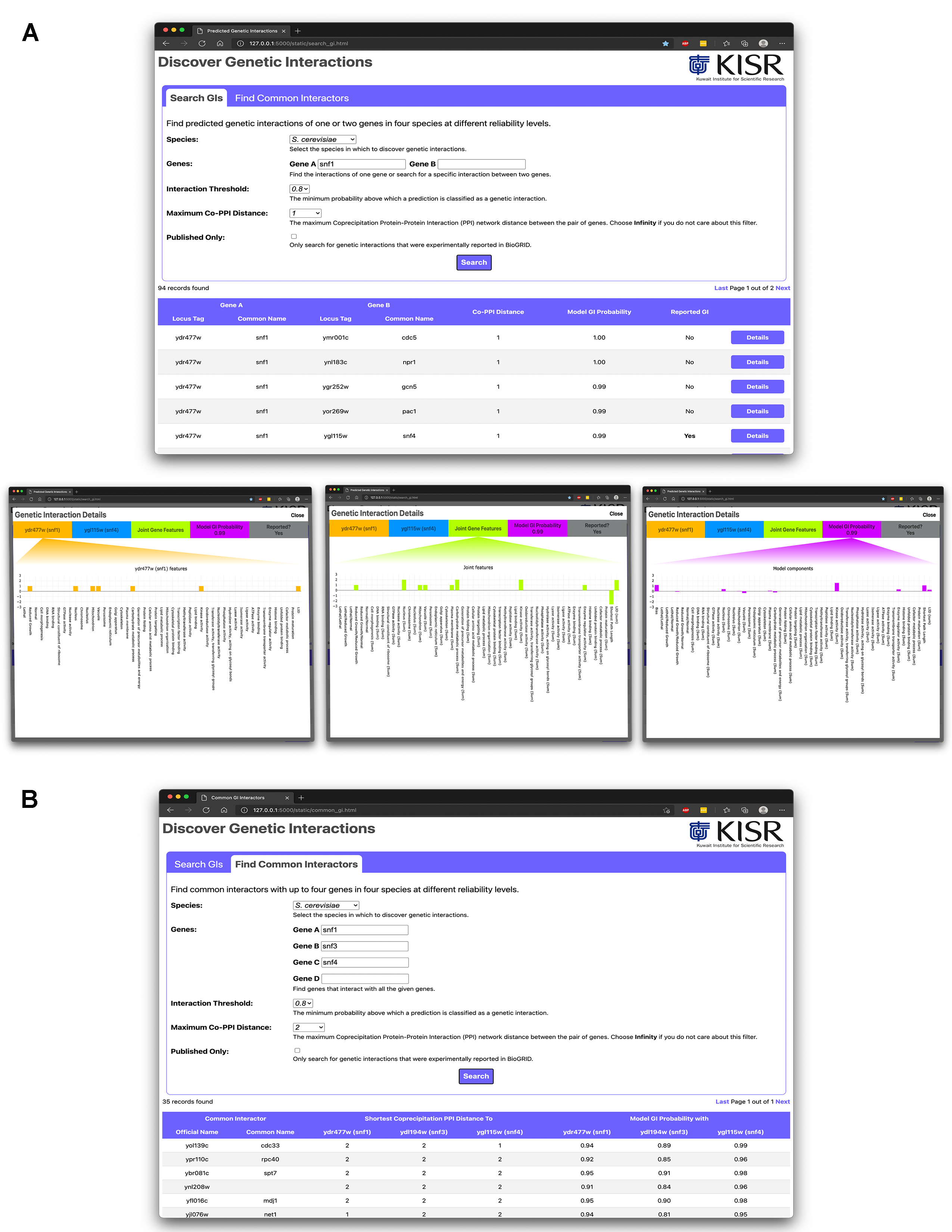

Supplement: btac519_Supplementary_Data [file btac519_supplementary_data.zip › btac519_Supplementary_Data/Supplementary Figure 13.tif]

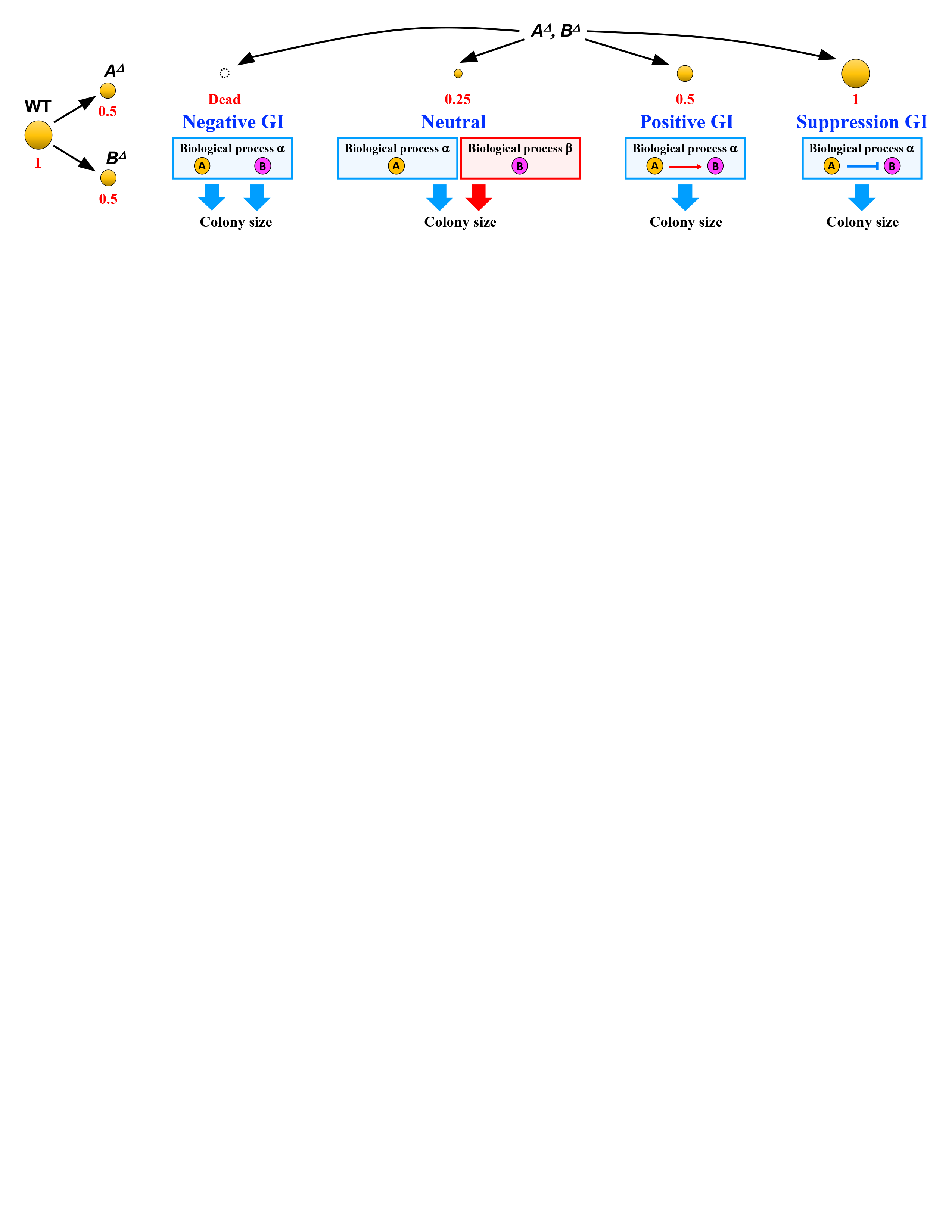

Supplement: btac519_Supplementary_Data [file btac519_supplementary_data.zip › btac519_Supplementary_Data/Supplementary figure 1B.tif]

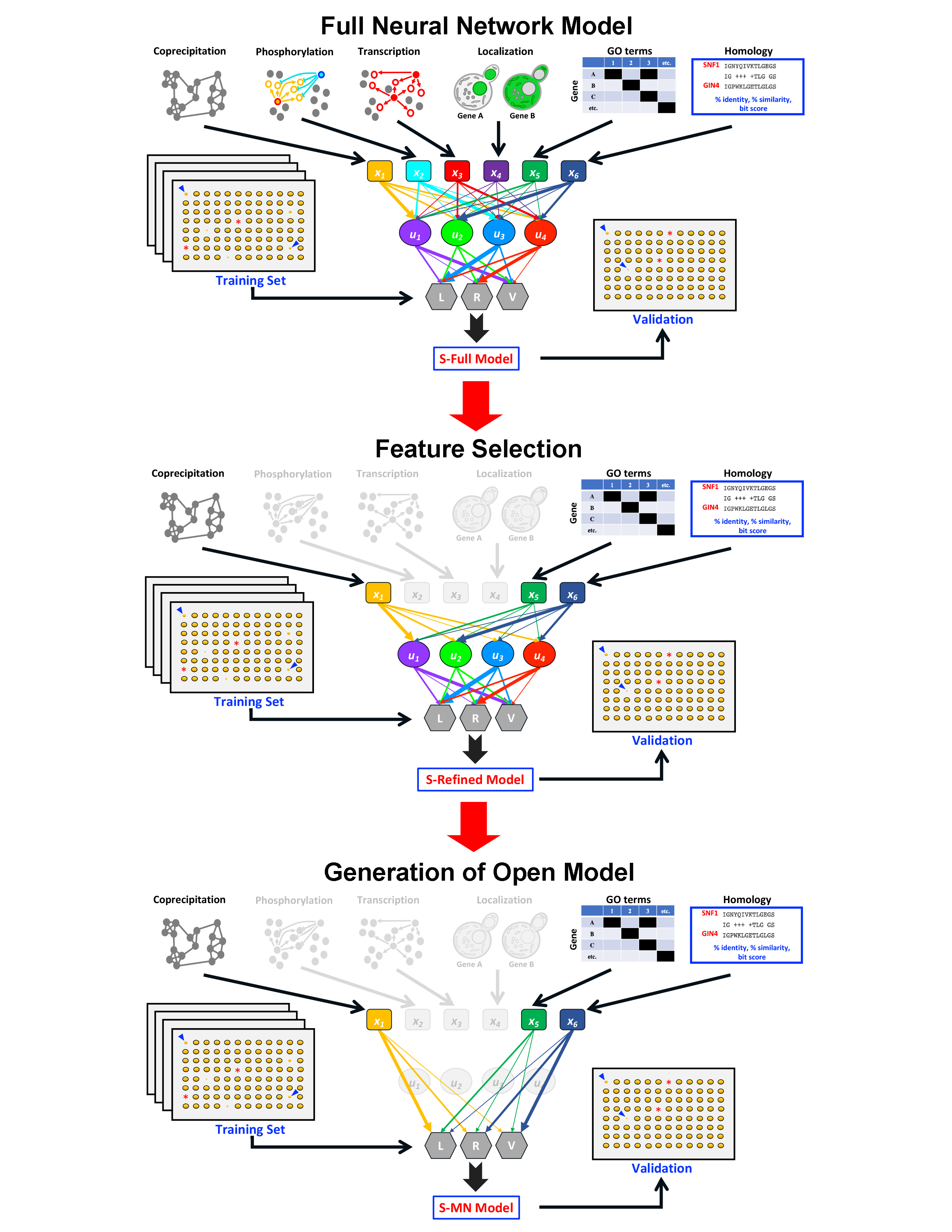

Supplement: btac519_Supplementary_Data [file btac519_supplementary_data.zip › btac519_Supplementary_Data/Supplementary figure 2B.tif]

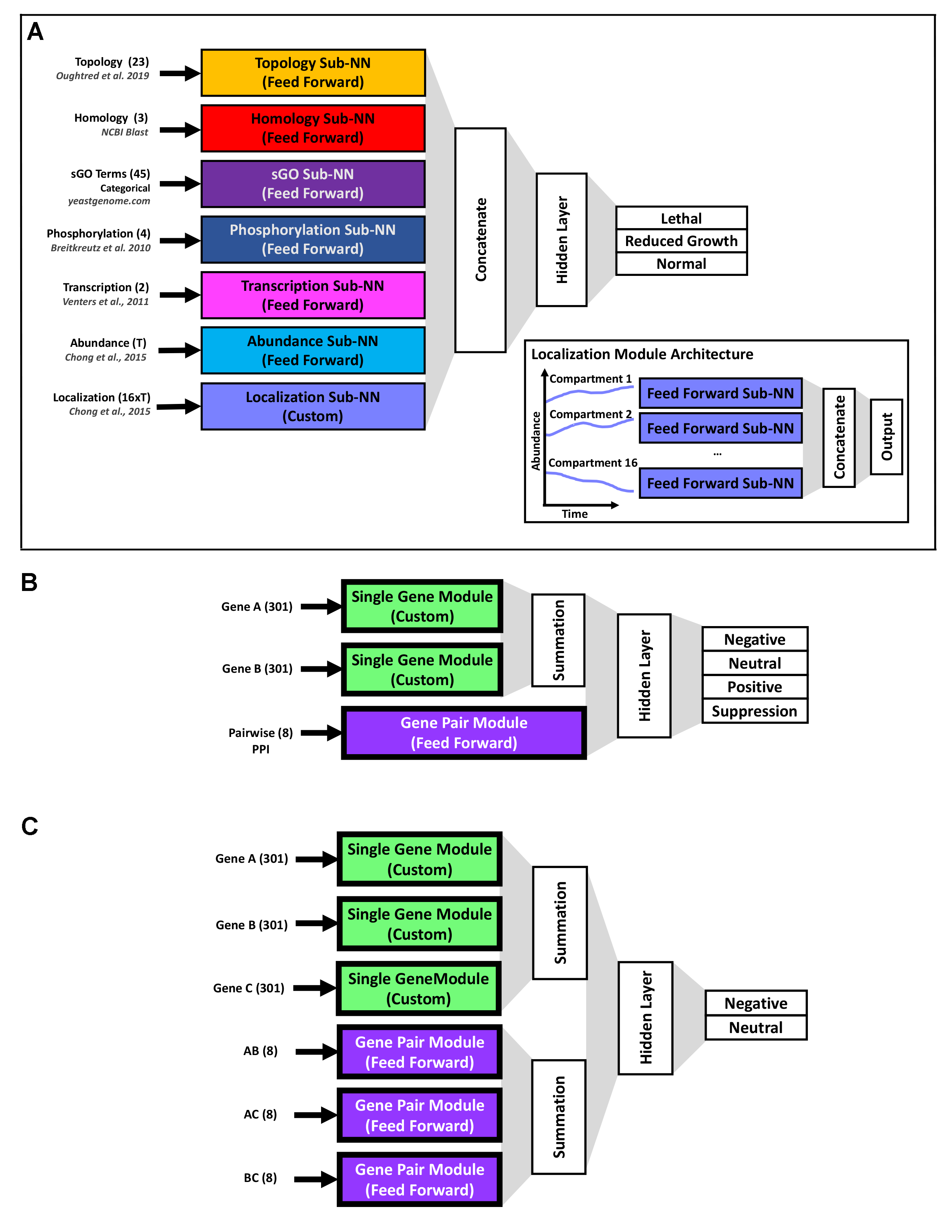

Supplement: btac519_Supplementary_Data [file btac519_supplementary_data.zip › btac519_Supplementary_Data/Supplementary figure 3B.tif]

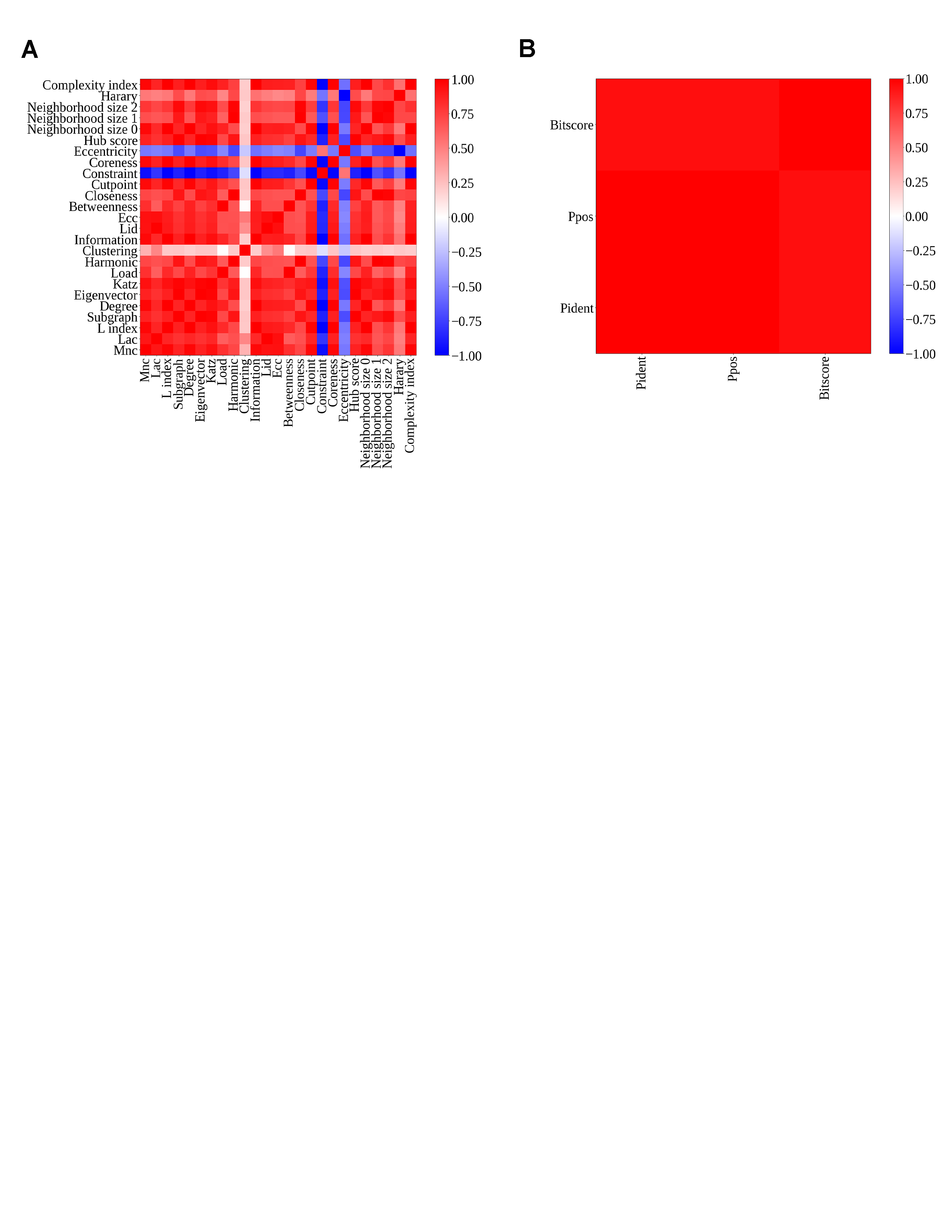

Supplement: btac519_Supplementary_Data [file btac519_supplementary_data.zip › btac519_Supplementary_Data/Supplementary figure 4B.tif]

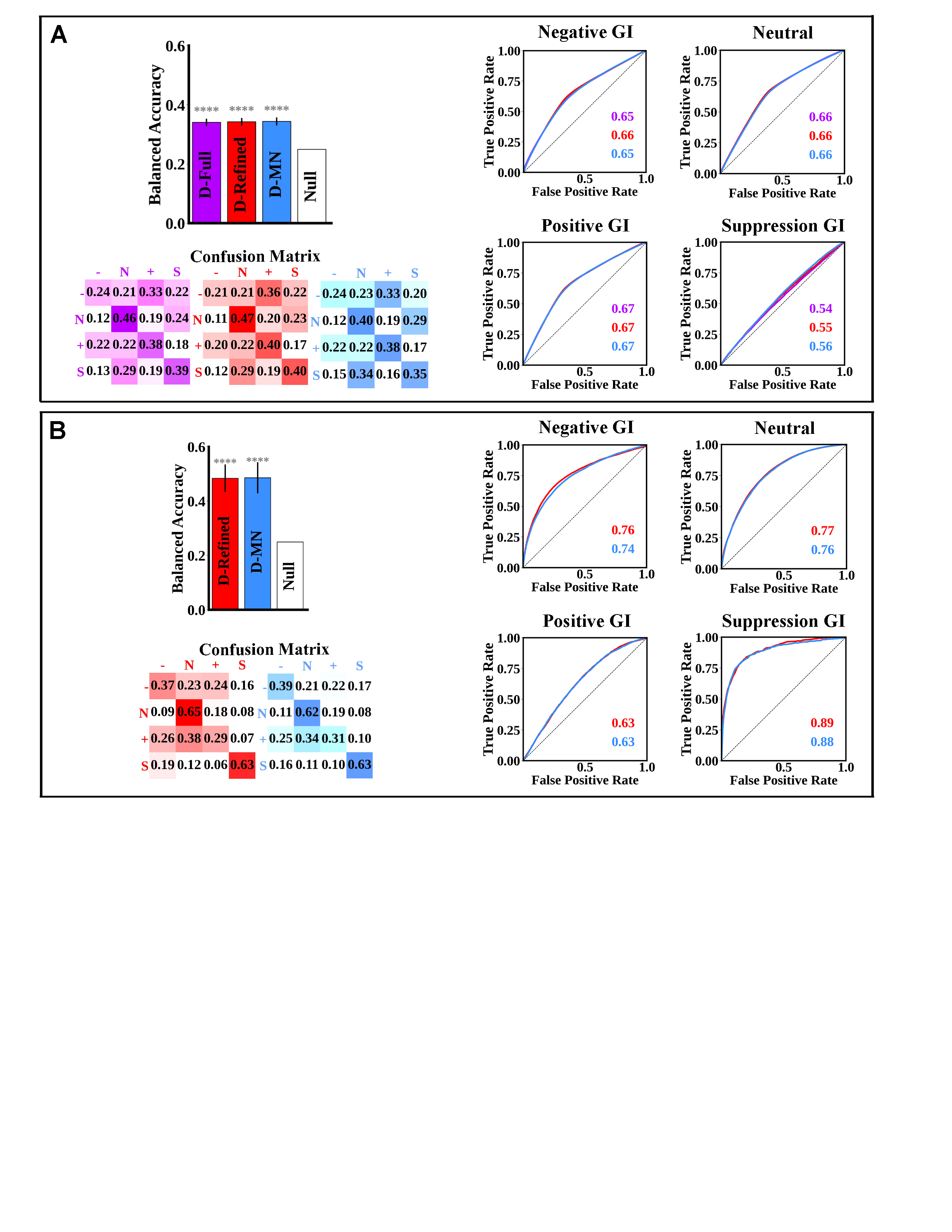

Supplement: btac519_Supplementary_Data [file btac519_supplementary_data.zip › btac519_Supplementary_Data/Supplementary figure 5B.tif]

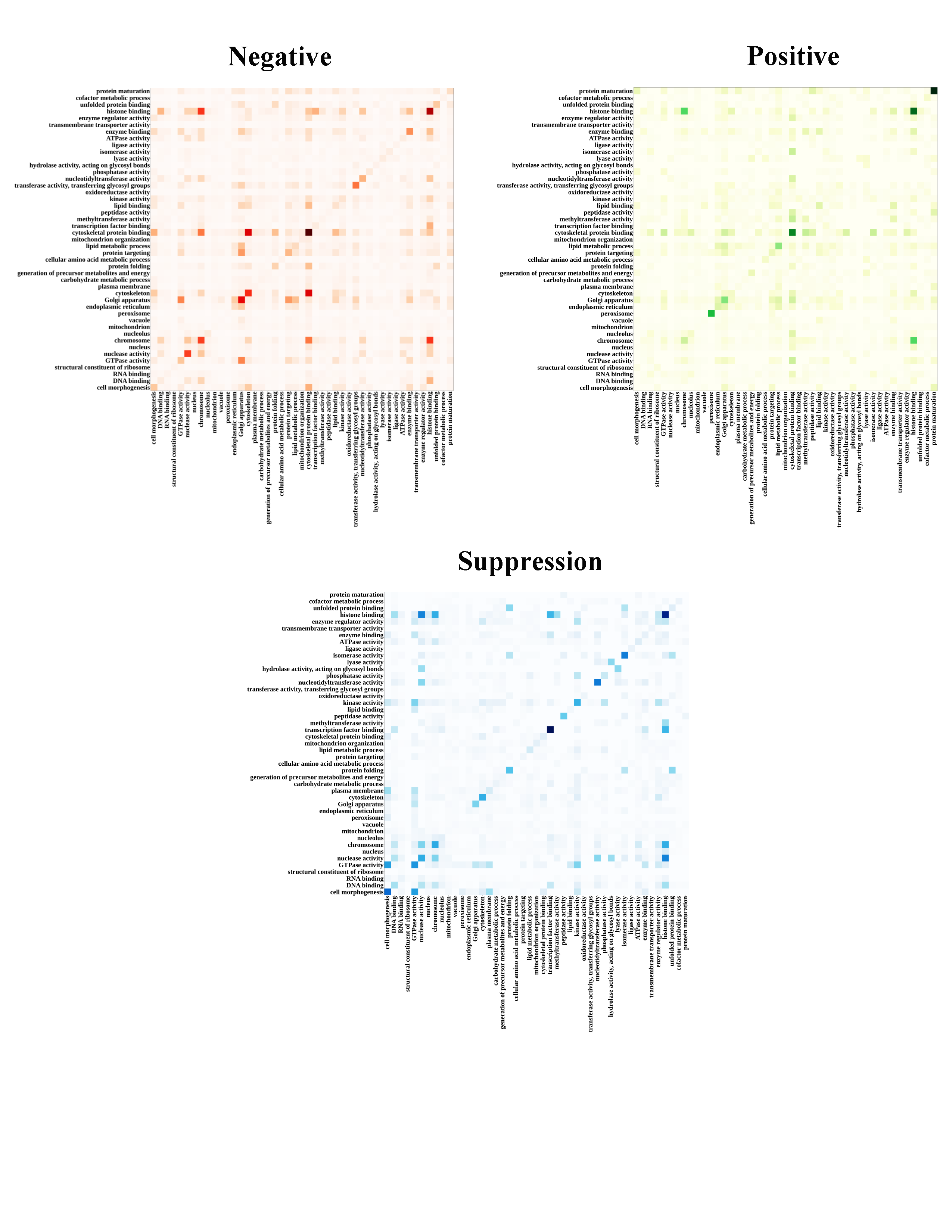

Supplement: btac519_Supplementary_Data [file btac519_supplementary_data.zip › btac519_Supplementary_Data/Supplementary figure 6B.tif]

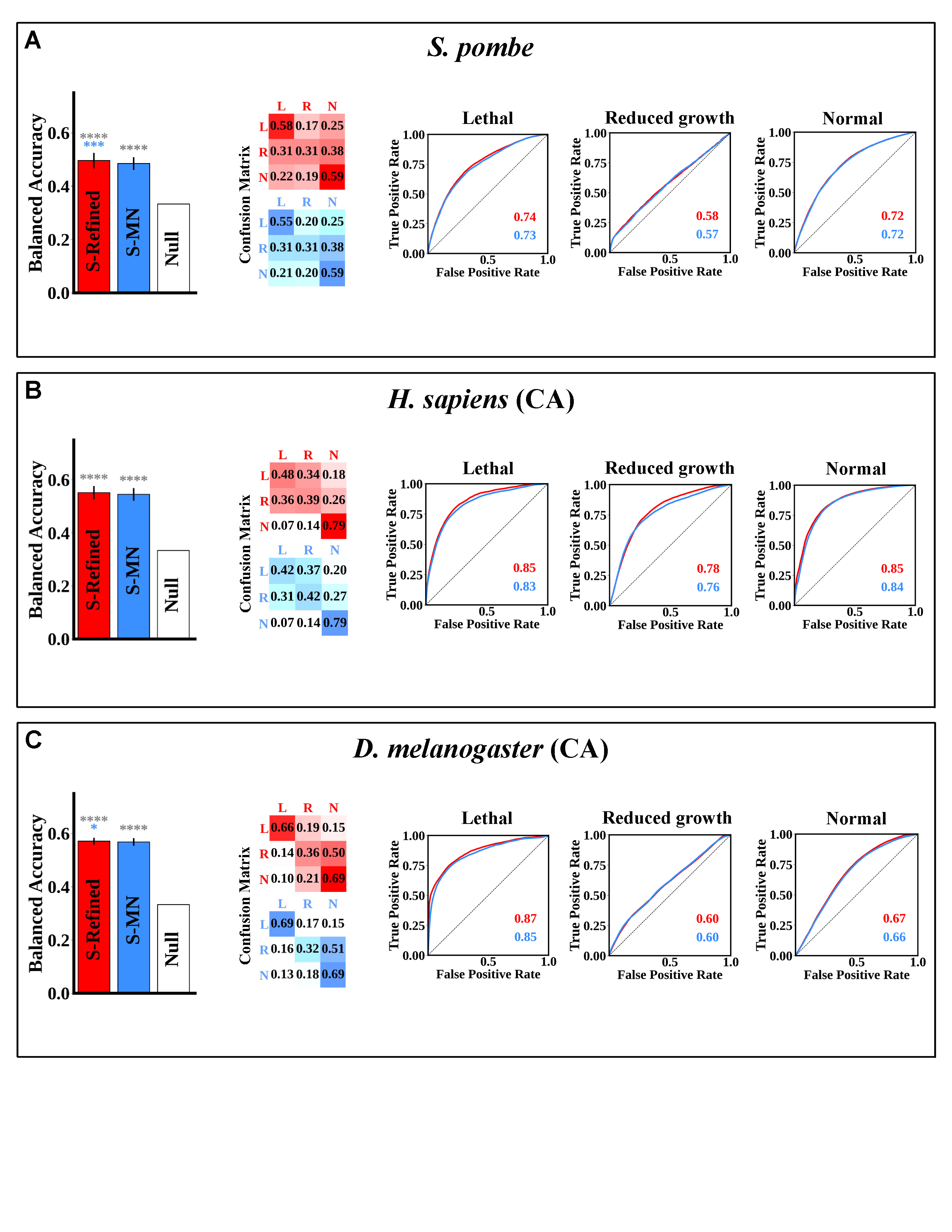

Supplement: btac519_Supplementary_Data [file btac519_supplementary_data.zip › btac519_Supplementary_Data/Supplementary Figure 7.tif]

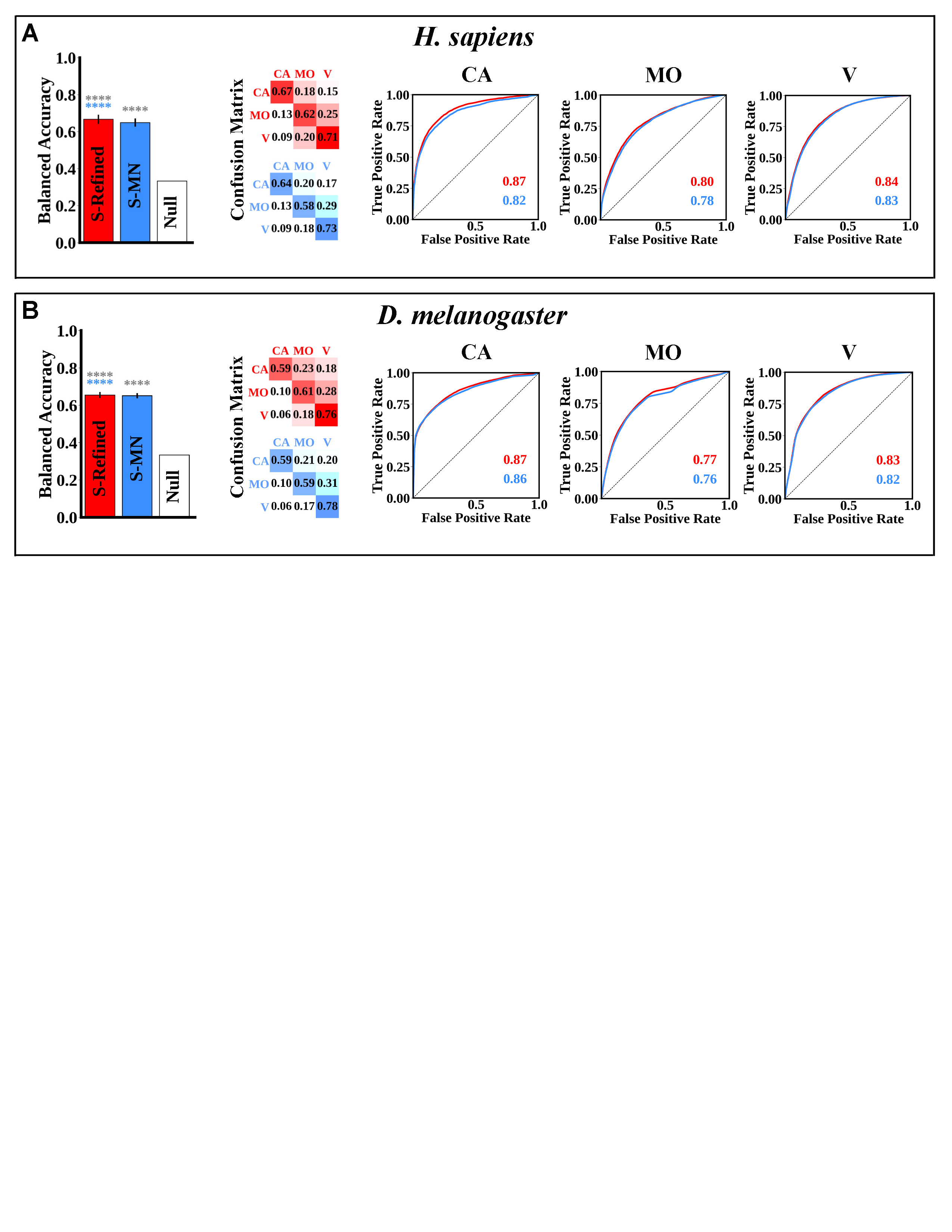

Supplement: btac519_Supplementary_Data [file btac519_supplementary_data.zip › btac519_Supplementary_Data/Supplementary Figure 8.tif]

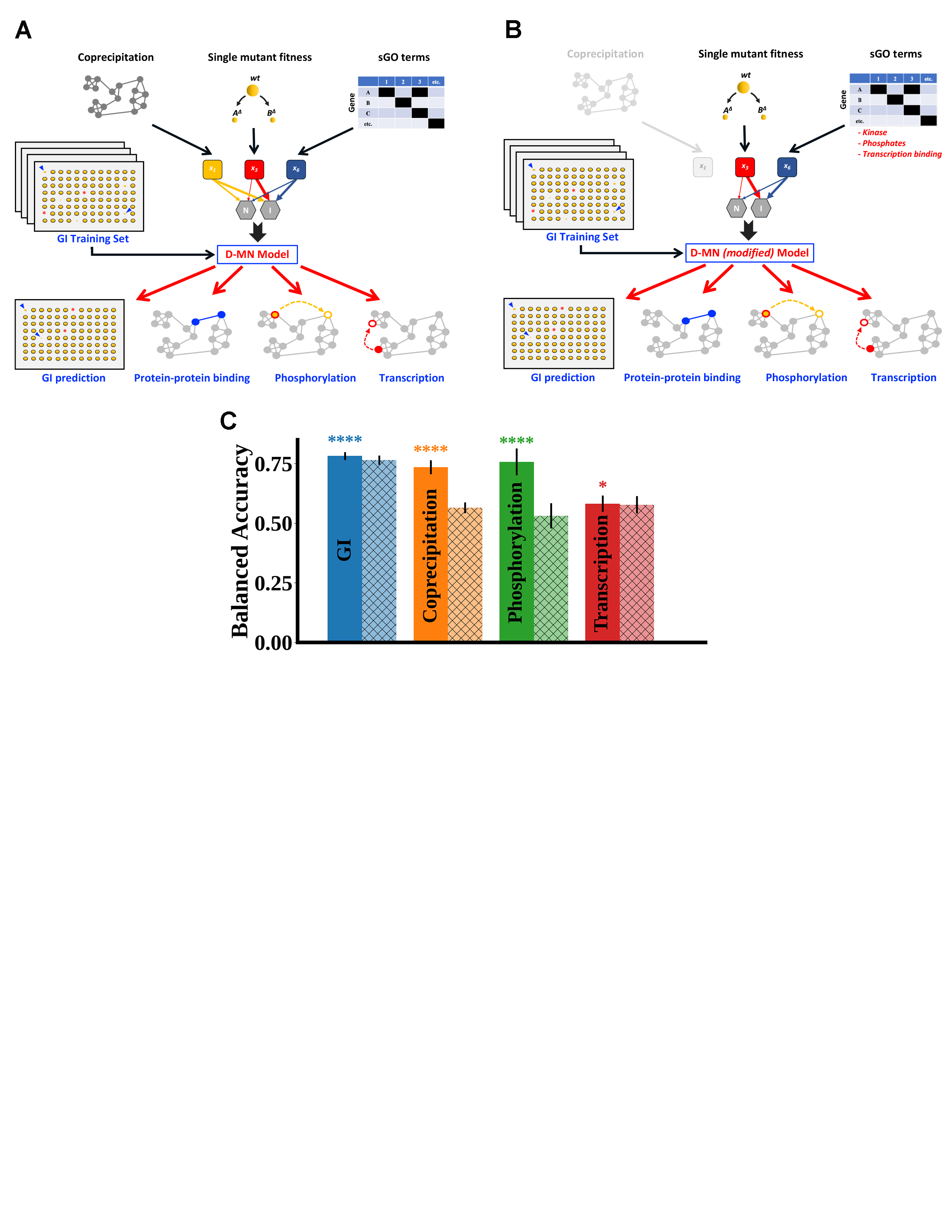

Supplement: btac519_Supplementary_Data [file btac519_supplementary_data.zip › btac519_Supplementary_Data/Supplementray Figure 10.tif]

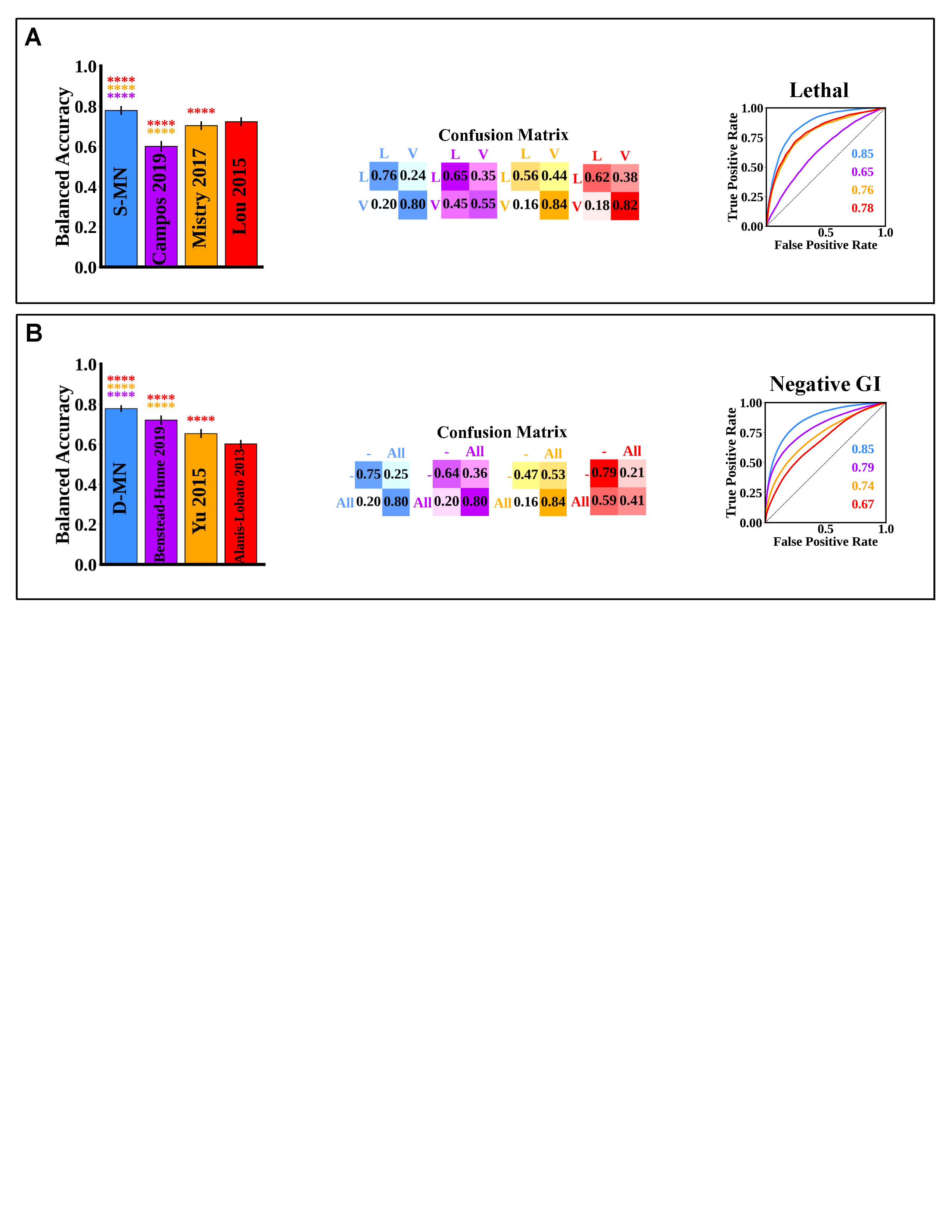

Supplement: btac519_Supplementary_Data [file btac519_supplementary_data.zip › btac519_Supplementary_Data/Supplementray Figure 9.tif]
